# Dynamically Allocated Hub in Task-Evoked Network Predicts the Vulnerable Prefrontal Locus for Contextual Memory Retrieval in Macaques

**DOI:** 10.1371/journal.pbio.1002177

**Published:** 2015-06-30

**Authors:** Takahiro Osada, Yusuke Adachi, Kentaro Miyamoto, Koji Jimura, Rieko Setsuie, Yasushi Miyashita

**Affiliations:** 1 Department of Physiology, The University of Tokyo School of Medicine, Hongo, Bunkyo-ku, Tokyo, Japan; 2 Department of Physiology, Juntendo University School of Medicine, Hongo, Bunkyo-ku, Tokyo, Japan; 3 Precision and Intelligence Laboratory, Tokyo Institute of Technology, Yokohama, Japan; 4 CREST, JST, Kawaguchi, Saitama, Japan; Oxford University, UNITED KINGDOM

## Abstract

Neuroimaging and neurophysiology have revealed that multiple areas in the prefrontal cortex (PFC) are activated in a specific memory task, but severity of impairment after PFC lesions is largely different depending on which activated area is damaged. The critical relationship between lesion sites and impairments has not yet been given a clear mechanistic explanation. Although recent works proposed that a whole-brain network contains hubs that play integrative roles in cortical information processing, this framework relying on an anatomy-based structural network cannot account for the vulnerable locus for a specific task, lesioning of which would bring impairment. Here, we hypothesized that (i) activated PFC areas dynamically form an ordered network centered at a task-specific “functional hub” and (ii) the lesion-effective site corresponds to the “functional hub,” but not to a task-invariant “structural hub.” To test these hypotheses, we conducted functional magnetic resonance imaging experiments in macaques performing a temporal contextual memory task. We found that the activated areas formed a hierarchical hub-centric network based on task-evoked directed connectivity, differently from the anatomical network reflecting axonal projection patterns. Using a novel simulated-lesion method based on support vector machine, we estimated severity of impairment after lesioning of each area, which accorded well with a known dissociation in contextual memory impairment in macaques (impairment after lesioning in area 9/46d, but not in area 8Ad). The predicted severity of impairment was proportional to the network “hubness” of the virtually lesioned area in the task-evoked directed connectivity network, rather than in the anatomical network known from tracer studies. Our results suggest that PFC areas dynamically and cooperatively shape a functional hub-centric network to reallocate the lesion-effective site depending on the cognitive processes, apart from static anatomical hubs. These findings will be a foundation for precise prediction of behavioral impacts of damage or surgical intervention in human brains.

## Abstract 2

Patterns of whole-brain activity while macaques perform a memory retrieval task show that the task-specific functional hub in the dynamic cortical network predicts the task-specific consequences of brain damage better than a task-invariant structural hub does.

## Author Summary

Patients with lesions in the front part of the brain’s frontal lobe—the prefrontal cortex—suffer from severe memory deficits. Neuroimaging and neurophysiology studies have revealed that multiple areas in the prefrontal cortex are activated during a specific memory task. However, the severity of the memory deficit after a lesion in the prefrontal cortex largely depends on which of the activated areas is damaged; lesions in only a fraction of the activated areas actually lead to memory deficits. It is currently unknown why some activated areas are “lesion effective” and others are not. Here, by using functional magnetic resonance imaging (fMRI) to measure macaque whole-brain activity during a memory task, we found that the activated areas and the task-specific functional connectivity among them formed a hierarchical network centered on a hub. The task-specific “functional hub” in this dynamic network accurately corresponds to the well-documented lesion-effective site and avoids the neighboring non-lesion-effective sites. Quantitatively, the predicted severity of memory impairment is proportional to the network “hubness” of the lesioned area in the functional network, rather than in the anatomical network, which is statically determined by axonal projection patterns. Our results suggest that the areas of the prefrontal cortex dynamically shape a hub-centric network, reallocating the lesion-effective site apart from the static anatomical hubs depending on the cognitive requirements of the specific memory task.

## Introduction

The prefrontal cortex (PFC) supports cognitive control of memory [1–6]. As shown using functional neuroimaging in humans, multiple PFC areas are activated in response to memory demands [4,6]. However, patients with typically broad cortical damage that includes a portion of these areas do not necessarily display memory deficits [1,2,7]: only a part of the activated areas is lesion effective, and the rest is not. Thus, it has been disputed whether the primary determinant of such a deficit is attributable to the individual brain areas subserving specific cognitive functions or to the information flow mediated by the connections among widely distributed PFC areas.

Recently, a third reconciliatory hypothesis was proposed suggesting that cortical areas form a highly ordered network containing hubs that play integrative roles in cortical information processing and lesioning the hub thus results in severe cognitive impairment [8,9]. Most investigations of the hub structure of the cortical network relied on structural neuroimaging by diffusion tractography reflecting anatomical connections or functional neuroimaging of spontaneous activity at rest [8,10], which was defined irrespective of any specific cognitive demand. In addition, the impact of abnormalities in such hubs has been examined in patients with nonfocal cortical pathologies, such as schizophrenia and Alzheimer disease [11–13]. Certainly, as implied by denser fiber tracts among distant brain centers in humans than in nonhuman primates [14], intellectual integrity spanning across multiple cognitive functions in humans would relate to strength of organic linkage among brain areas in hardwired anatomical network structure or in effect relate to hubness of each area in the network. However, the lesion-effective site for a specific cognitive task is historically known to vary depending on the corresponding cognitive process, and impairment of a specific function is not always accounted for by structural network hubs. For example, in semantic processing (retrieving and selecting among competing alternatives), patients with lesions in the superior frontal gyrus, which is suggested to be a structural hub area in the whole brain network delineated by diffusion tensor imaging [15], did not show impairment, whereas patients with lesions in the inferior frontal gyrus, which is not a structural hub area [15], showed severe impairment [16]. Accordingly, the structural network-based approach is not versatile enough to localize a vulnerable locus for a task-specific cognitive function, damage of which would impair performance of the task.

Here, we hypothesized that task-activated PFC areas form an ordered network centered at a task-specific “functional hub,” and the lesion-effective site corresponds to the “functional hub,” but not to a task-invariant “structural hub” in a static anatomical network. We tested this functional hub hypothesis using a memory task requiring retrieval of the temporal context of past events [17–23]. Performance of this behavioral task is known to be impaired after damage to the lateral PFC in humans [17]. In macaques, this contextual memory retrieval is impaired by experimentally controlled focal lesions of the mid-dorsolateral PFC (MDL-PFC), but not by lesions of the periarcuate area [18]. Therefore, we recorded whole brain activity in awake macaque monkeys via functional magnetic resonance imaging (fMRI) [24–36] while the animals performed the contextual memory retrieval task, and measured PFC activity and task-evoked directed connectivity among the PFC areas during successful retrieval. Task-evoked connectivity calculated by a psychophysiological interaction (PPI) analysis is a suitable index to evaluate task-specific changes in the relationship between brain areas [37]. Because abundant information regarding directed axonal projections in macaques is available from previous tracer studies [38,39], we were also able to examine the “hubness” of each PFC area in terms of the directed anatomical network, which can be investigated only in macaques, but not in humans.

From the identified task-evoked connectivity network, we estimated the behavioral impact of lesioning each activated area by using a novel simulated-lesion method. The predicted severity of impairment from this method accorded well with the areal dissociation in contextual memory impairments observed after lesions to different macaque PFC areas [18]. Quantitatively, we demonstrated that the predicted severity of impairment was proportional to the functional network hubness (betweenness centrality; Box 1) of the virtually lesioned area, which was calculated based on interareal directed task-evoked connectivity. By contrast, the predicted severity was not explained by the anatomical network hubness calculated based on interareal directed anatomical connections. Taken together, these results support our functional hub hypothesis and suggest that PFC areas dynamically and cooperatively shape a hub-centric network to reallocate the lesion-effective site depending on the cognitive processes, apart from anatomical hubs.

**Box 1. Glossary**

**Betweenness centrality:** a measure of the importance of a node within a network architecture, indicating how much the node interacts with all the other nodes; it is calculated as the fraction of all shortest paths in the network that contain a given node [8,12,40].

**Task-evoked connectivity:** a functional connectivity between one region and another that is modulated by task demand; in this manuscript, we used PPIs as task-evoked connectivity [37].

**Anatomical connectivity:** connectivity between regions through axonal projections, the existence of which was detected by using antero- or retrograde tracing methods; CoCoMac database is a collection of past tracer studies in the macaque cerebral cortex [41].

**Functional hub:** a region occupying a central position in a network determined by the task-evoked connectivity; a region with high betweenness centrality in the task-evoked connectivity network.

**Anatomical hub:** a region occupying a central position in a network determined by the anatomical connectivity; a region with high betweenness centrality in the anatomical connectivity network [8].

**Hub-centric network:** a network within which information transmission is supported by a limited numbers of hub regions; a network has hub regions with statistically significant betweenness centrality.

**Hubness:** tendency of a region to play a key role in information transmission within a network; in this manuscript, hubness is evaluated by betweenness centrality.

**Outward/inward connectivity:** task-evoked connectivity from a given region to other regions (outward connectivity) or to a given region from other regions (inward connectivity); note that explicit correspondence with anatomical connectivity (anterograde/retrograde connection) does not exist (see Discussion) [37,42].

**Homotopic regions:** pairs of geometrically corresponding interhemispheric brain regions.

**Lesion-effective site:** a brain site, lesions of which induce severe behavioral impairment in a specific task.

## Results

### Temporal-Order Memory Retrieval in Macaques

**Fig 1:**
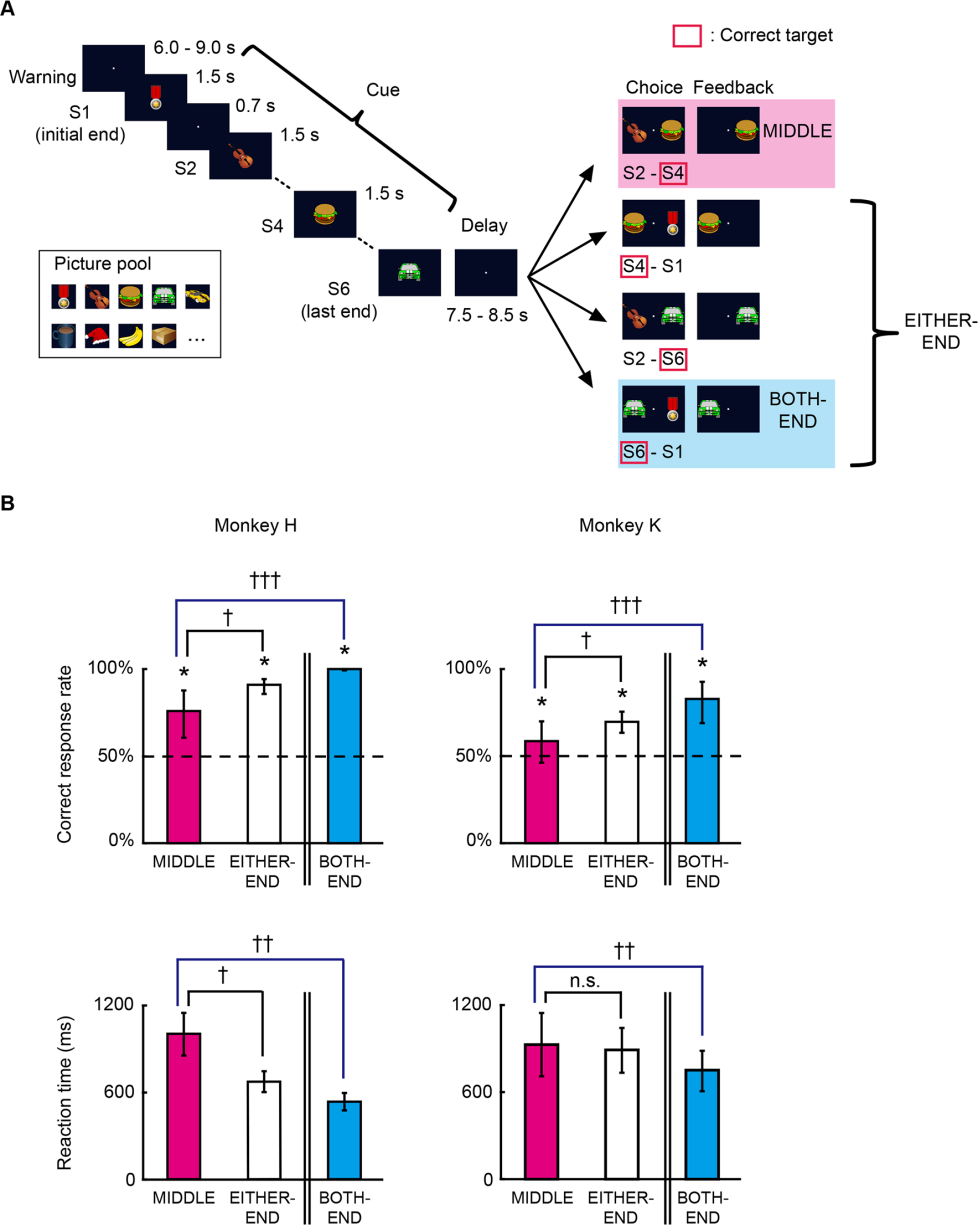
Temporal-order judgment task and behavioral performance of monkeys. (A) Trial structure in the temporal-order judgment task. In each trial, monkeys pulled a joystick to initiate the trial (Warning), after which a list of stimuli was presented serially (Cue). After a delay (Delay), two stimuli from the list were presented simultaneously (Choice). The monkeys were required to select the stimulus that had been presented more recently in the list. The time parameters and trial structure for monkey H are shown here. The stimuli were selected from a 1,200-picture pool of natural and artificial objects (from Microsoft Clip Art or HEMERA Photo-Object database) in a pseudorandom order. The images provided in this figure are representations only and were not used in the experiment. (B) Percentages of correct responses (upper) and reaction times (lower) for each monkey during scanning sessions. The dashed line indicates the chance level. Error bars indicate standard deviation (SD) across sessions. * *p* < 10^−4^, *t*-test. † *p* < 10^−4^, †† *p* < 10^−5^, ††† *p* < 10^−7^, paired *t*-test.

We conducted fMRI in two macaque monkeys (*Macaca mulatta*) performing a temporal-order judgment task with a list of visual stimuli (Fig 1A). In this task, after serial presentation of a stimulus list (Cue), the monkeys were simultaneously presented with two of the listed stimuli and were required to judge which stimulus had been presented more recently (Choice). In the judgment, two types of trials were performed: trials in which the stimulus pair in Choice included neither the initial nor the last end stimuli in the list (MIDDLE trial) and trials in which at least one of the paired stimuli was an end (initial or last) stimulus in the list (EITHER-END trial). Among the EITHER-END trials was a trial in which the stimulus pair consisted of both an initial and last end stimuli (BOTH-END trial) (Fig 1A). The correct response rate for each trial type significantly exceeded chance for both monkeys (all *p* < 10^−4^, *t*-test) (Fig 1B, upper panels and S1 Fig, upper panels). Significant differences in correct response rates and reaction times among the trial types were indicative of differences in the demand for temporal-order judgment due to the inclusion of end stimuli, particularly in BOTH-END trials (all *p* < 10^−4^, paired *t*-test for each monkey, except *p* = 0.12 for reaction times between MIDDLE and EITHER-END trials in monkey K) (Fig 1B and S1 Fig). These results are consistent with those of earlier studies with temporal-order judgment in humans [17,21] and monkeys [18]. The contrast of “MIDDLE minus BOTH-END” was used to identify the neural correlates of contextual memory retrieval in the present fMRI study because the BOTH-END condition captured more homogeneous cognitive components compared with the EITHER-END condition (i.e., the last end stimulus in the list was chosen in every trial). We also compared the eye movement data during temporal-order judgment. No significant differences were detected in eye traces between MIDDLE and BOTH-END conditions (for average eye position, *p* > 0.15, *t*-test; for fluctuation of eye positions, *p* > 0.3, two-way analysis of variance [ANOVA]; see “Data Analysis of Eye Movement” in S1 Text).

### Identification of Cortical Regions Active for Temporal-Order Retrieval

**Fig 2:**
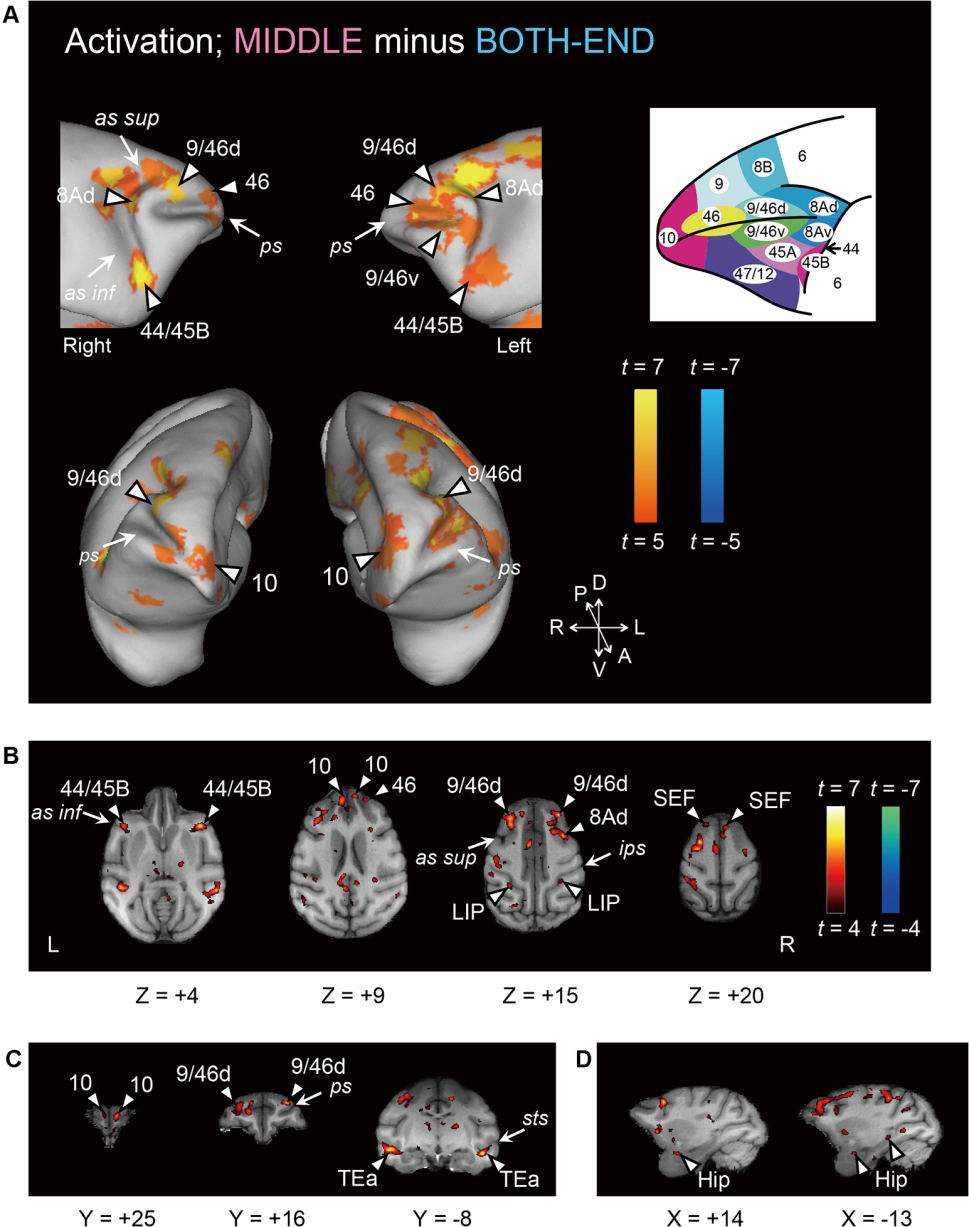
Brain regions active for temporal-order judgment. (A) Activity related to temporal-order judgment revealed by the contrast of MIDDLE minus BOTH-END. An activation map is superimposed on the inflated brain: top, lateral view; bottom, anterior view. Inset shows the atlas of the macaque prefrontal cortex based on Petrides (2005) [4] and Petrides (1994) [46]. *ps*, principal sulcus; *as sup*, superior branch of arcuate sulcus; *as inf*, inferior branch of arcuate sulcus. (B–D) Activation map is superimposed on transverse sections (B), coronal sections (C), and sagittal sections (D). LIP, lateral intraparietal area; SEF, supplementary eye field; Hip, hippocampus; *ips*, intraparietal sulcus; *sts*, superior temporal sulcus.

Comparison of fMRI signals in MIDDLE and BOTH-END conditions revealed significant activation of multiple cortical areas (Fig 2; Table 1, *p* < 0.05, corrected for multiple comparisons based on family-wise error [FWE]). The majority of the significant bilateral activations were located in the PFC (Fig 2A–2C). Significant bilateral activations were found in the frontopolar area (area 10; see inset in Fig 2A for areal nomenclature and demarcation). Bilateral activations were also found in the dorsolateral surface and dorsal bank of the principal sulcus (area 9/46d). Lesioning this area was previously reported to impair temporal-order judgment [18]. Interestingly, activations were found in the periarcuate area (area 8Ad), though lesions in that area produce no behavioral deficit [18]. In the temporal cortex, the bilateral activations were found in the ventral bank of the superior temporal sulcus (area TEa) (Fig 2C). In the parietal cortex, activations were found in the lateral bank of the intraparietal sulcus (lateral intraparietal area [LIP]) (Fig 2B). In the medial temporal regions, bilateral hippocampi were activated (Fig 2D), where the involvement in memory on temporal context has been suggested both in humans [43,44] and monkeys [45]. We focused on ten “homotopic” areas (see Materials and Methods) with bilateral significant activation in both monkeys (S1 Table). For activation patterns among the ten homotopic areas, there was a significant correlation between monkeys (*r* = 0.25, *p* = 0.004). To confirm that these activations were not due only to the differences in reaction times between the MIDDLE and BOTH-END trials (Fig 1B), we conducted a reaction time-corrected analysis using parametric modulation (see Materials and Methods). Homotopic activations detected in the original analysis, including the multiple PFC spots, still remained significant after the reaction time correction (S2 Table). Comparison of fMRI signals in MIDDLE and EITHER-END conditions also showed significant activations of multiple cortical areas (S2 Fig). All the homotopic activations in the original analysis remained significant (S3 Table).

**Table 1:** Brain regions activated in MIDDLE minus BOTH-END contrast.

	Coordinates (mm)					
Hemisphere	X	Y	Z	*t* value	Area	
**Frontal**						
L	-3	23	10	5.93	10	*
R	3	25	9	5.55		
L	-11	18	12	5.87	46	*
R	9	22	11	5.76		
L	-14	15	12	5.58	9/46v	*
R	16	15	10	4.05‡		
L	-12	14	15	6.01	9/46d	*
R	12	16	15	6.19		
L	-19	10	4	5.46	44/45B	*
R	18	10	3	6.64		
L	-6	11	21	5.51	SEF	*
R	3	9	20	5.01†		
L	-11	10	17	6.78	8Ad	*
R	10	13	16	5.54		
L	-16	12	10	6.40	9/46v post	#
R	14	6	15	6.16	8Ad post	#
L	-18	9	12	5.78	8Av	#
R	1	6	21	6.03	SMA ant	#
L	-2	-1	21	5.91	SMA post	#
L	-10	2	20	6.09	PMd ant	#
L	-11	-1	20	6.22	PMd post	#
L	-20	6	7	5.99	PMv	#
L	-7	15	10	6.09	24c ant	#
L	-3	1	14	6.08	24c post	#
**Parietal**						
L	-12	-19	17	6.00	LIP	*
R	13	-16	15	4.51†		
L	-18	-9	17	6.24	5	#
L	-12	-12	22	6.00	S1	
L	-3	-14	10	7.57	30	#
L	-2	-20	9	5.65	23b	#
**Temporal**						
L	-24	-8	-8	6.31	TEa	*
R	22	-8	-10	6.50		
R	18	2	-13	5.77	TEa ant	#
R	17	1	-9	6.21	TPO	#
R	19	-2	-10	6.04	PGa	
L	-26	-7	-10	5.57	TEpd	#
L	-21	-20	4	6.10	TEO	#
R	10	-17	-4	5.67	TFO	#
L	-20	-1	-4	5.81	Insula ant	#
L	-21	-4	-1	5.80	Insula post	#
**Occipital**						
R	26	-21	4	6.19	V4	#
R	24	-23	-1	6.15	V4	#
R	24	-23	8	5.86	V4	#
L	-18	-30	2	5.70	V3v	#
**Hippocampus**						
L	-13	-2	-10	4.91†	Hip	*
R	14	-1	-10	5.17†		
L	-13	-19	-2	4.68†	Hip post	
R	16	-9	-14	4.34†	Hip mid	
**Others**						
R	3	-14	2	6.27	Thalamus	#
R	8	9	6	5.76	Cd	#
R	14	9	1	5.73	Cd	#
R	5	-16	-4	7.13	SC	#
L	-9	-20	-8	5.44	Cerebellum	#

Significant peaks at a voxel level of *p* < 0.05 corrected by FWE. Coordinates are listed in monkey bicommissural space [26,28,34].

† Significant only at a voxel level of *p* < 0.001 corrected by false discovery rate (FDR).

‡ Significant only at a voxel level of *p* < 0.005 corrected by FDR.

* Homotopic area.

# Nonhomotopic area but the area used for the PPI analysis with a larger set of areas in S10 Fig (see Materials and Methods).

10, area 10; 46, area 46; 9/46v, area 9/46 ventral part; 9/46d, area 9/46 dorsal part; 44/45B, area 44/45B; SEF, supplementary eye field; 8Ad, area 8A dorsal part; 8Av, area 8A ventral part; SMA, supplementary motor area; PMd, dorsal premotor area; PMv, ventral premotor area; 24c, area 24c; LIP, lateral intraparietal area; 5, area 5; S1, primary somatosensory cortex; 30, area 30; 23b, area 23b; TEa, area TEa; TPO, area TPO; PGa, area PGa; TEpd, area TEpd; TEO, area TEO; TFO, area TFO; V4, visual area 4; V3v, visual area 3 ventral part; Hip, hippocampus; Cd, caudate nucleus; SC, superior colliculus; ant, anterior; post, posterior; mid, middle.

### Hub-Centric Prefrontal Cortical Network

**Fig 3:**
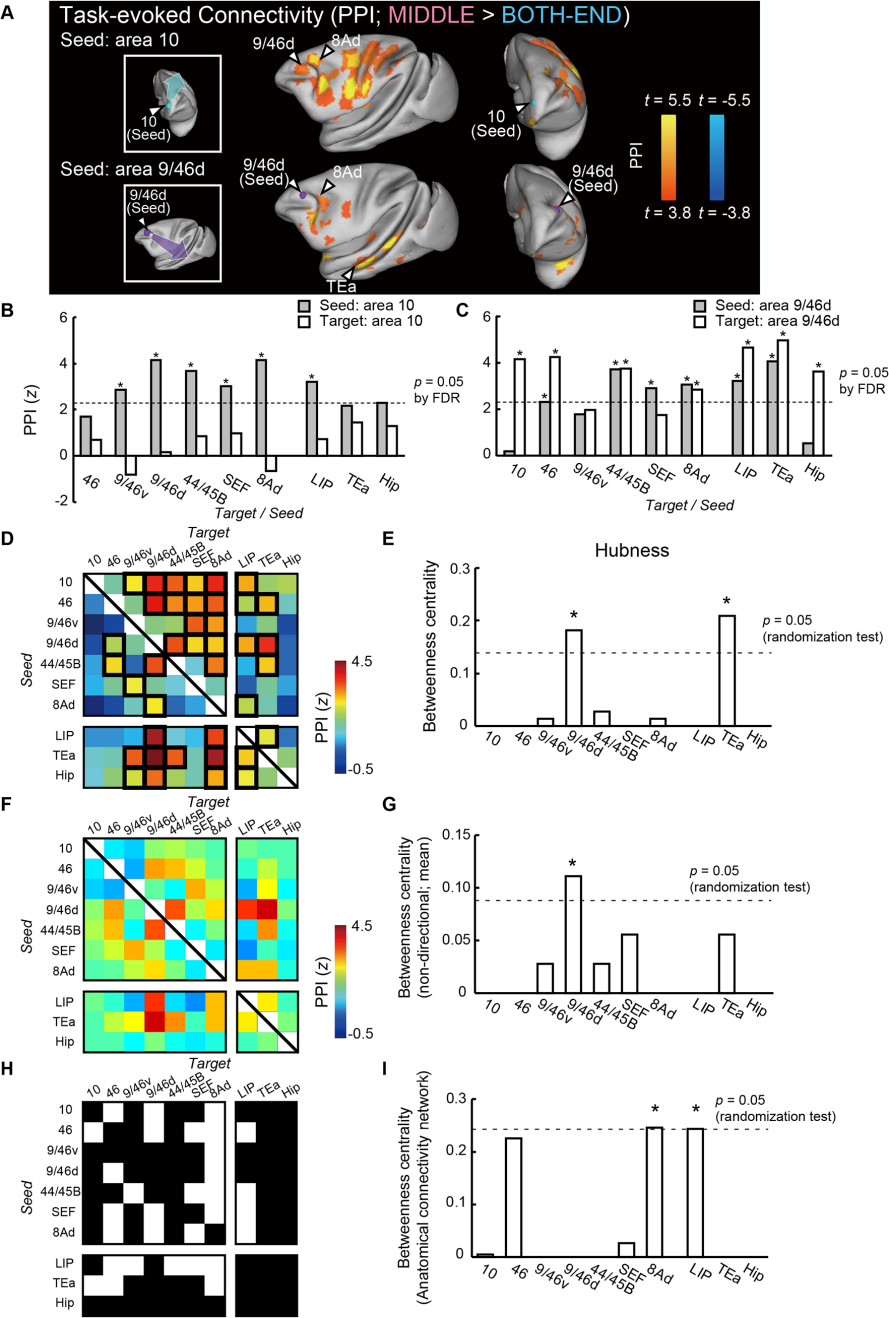
Hub-centric cortical network for temporal-order judgment. (A) PPI (MIDDLE > BOTH-END). Color *t*-map of PPI is superimposed on the inflated brain. Upper and lower panels show the PPI maps for the seeds in areas 10 and 9/46d, respectively. (B, C) Two bar plots in each column show *z*-values for PPIs from area 10 (B) or area 9/46d (C) to other ipsilateral homotopic areas (gray) and PPIs from other homotopic areas to area 10 (B) or area 9/46d (C) (white). Dashed lines indicate significant *z*-value (*p* = 0.05 [FDR correction]). * *p* < 0.05 (FDR correction). (D) PPI matrix among the ten homotopic areas. Rows and columns indicate seed and target areas, respectively. Significant connectivities are enclosed by thick black lines (*p* < 0.05 [FDR correction]). (E) Betweenness centralities of each area calculated based on (D). The dashed line indicates the significance at *p* = 0.05 (randomization test [comparison with the distribution of the randomized network]). * *p* < 0.05. (F) PPI matrix among the ten homotopic areas without assumptions of directionality. The weight of the connection between A and B is evaluated as the mean value of PPI_*A->B*_ and PPI_*B->A*_. (G) Betweenness centralities of each area calculated based on (F). The dashed line indicates significance at *p* = 0.05 (randomization test). * *p* < 0.05. (H) Anatomical connectivity matrix among the ten homotopic areas. Rows and columns indicate seed and target areas, respectively. A white (black) square indicates the presence (absence) of anatomical connection from row to column. Anatomical information is based on the CoCoMac database [41,47,48]. The projections to/from areas 8Ad, SEF, and LIP listed in the matrix are categorized as those to/from areas 8A, 6DR, and POa in CoCoMac, respectively. (I) Betweenness centralities of each area calculated based on (H). The dashed line indicates significance at *p* = 0.05 (randomization test). * *p* < 0.05.

We next conducted a PPI analysis to examine whether temporal-order retrieval load affects connectivity among the identified areas. When we located the PPI seed in area 10, a significant increase in task-evoked connectivity (MIDDLE > BOTH-END) from this area was found in diverse PFC areas, including areas 9/46d and 8Ad (Fig 3A, upper panels). On the other hand, when we located the PPI seed in area 9/46d, a significant increase in task-evoked connectivity was found in areas 8Ad and TEa, but not in area 10 (Fig 3A, lower panels; for the profiles of PPI values for areas 10 and 9/46d, Fig 3B and 3C, S3 Fig; for the characterization of functions of area 10, see S4 Fig). When we estimated all the combinations of PPIs among the ten homotopic areas within the same hemisphere, a three-way ANOVA on PPI values (laterality [left or right] × seed area × target area) revealed a significant interaction between seed area and target area (*F*(81, 81) = 1.49, *p* = 0.03) with no significant main effect of laterality (*F*(1, 1) = 0.001, *p* = 0.98) or its interactions with seed area (*F*(9, 9) = 0.45, *p* = 0.87) or with target area (*F*(9, 9) = 0.73, *p* = 0.67). These results indicate that the PPI patterns were characterized solely by combinations of connectivity among the homotopic areas (Fig 3D). Similar PPI patterns among the ten homotopic areas were found in the PPI connectivities with contralateral regions (*r* = 0.78, *p* = 1.1 × 10^−19^) (S5 Fig; for individual monkey data, S6 Fig), and these patterns were significantly correlated between monkeys (*r* = 0.25, *p* = 0.004).

In order to detect a functional “hub” in the network of 90 (i.e., 10 × (10–1)) possible connections among the ten homotopic areas (Fig 3D), the network metric “betweenness centrality” (fraction of shortest paths passing through a certain node [area]) [8,12] was calculated. The betweenness centrality of area 9/46d was the highest among the PFC areas, and areas 9/46d and TEa were the only areas with statistically significant betweenness centralities (*p* = 0.009 for area 9/46d, *p* = 0.002 for area TEa, randomization test [comparison with the distribution of randomized network]; see S7 Fig, Materials and Methods) (Fig 3E). In the binarized network (thresholded at *p* < 0.05 with false discovery rate [FDR] correction) (Fig 3D), both the degree (total number of connections possessed by an area) and the betweenness centrality of area 9/46d were significant and the highest among the ten homotopic areas (*p* = 0.001 for degree, *p* = 0.01 for betweenness centrality, randomization test) (S8 Fig). Even without any assumptions of directionality in PPI, the betweenness centrality of area 9/46d was confirmed to be significant (all *p* < 0.05, randomization test) (Fig 3F and 3G, S9 Fig; see Materials and Methods). Therefore, area 9/46d makes the strongest contribution to communication within the contextual memory retrieval network via the shortest paths among the homotopic areas and thus functions as a “hub.” Comparisons between the profiles of betweenness centrality values calculated based on the directional and nondirectional PPI matrices demonstrate that the hub structure determined by PPI network (area 9/46d) is sufficiently robust not to be affected by the directional effects of the PPI. The outstanding betweenness centrality of area 9/46d was also confirmed in a network containing a larger set of areas that included nonhomotopic areas (total of 39 areas; S10 Fig; see Materials and Methods). Hence, the cortical areas contributing to contextual memory retrieval cooperatively form an ordered network centered at area 9/46d as a functional hub.

We also evaluated the directed axonal projection pattern among the activated areas with the aid of CoCoMac database (collection of past tracer studies in the macaque cerebral cortex) (Fig 3H, S11A Fig) [41,47,48]. Based on the network of anatomical connections among the ten homotopic areas (Fig 3H), the betweenness centralities of areas 8Ad and LIP were statistically significant (*p* = 0.04 for area 8Ad, *p* = 0.04 for area LIP, randomization test), whereas that of area 9/46d was not (*p* > 0.9, randomization test) (Fig 3I). In the anatomical network containing a larger set of areas that included nonhomotopic areas (S11A Fig), areas 8Ad and LIP, along with areas 46, 24c, 23b, TFO, and thalamus, were also confirmed to have significant betweenness centralities (all *p* < 0.05, randomization test), whereas area 9/46d did not have a significant betweenness centrality (*p* > 0.9, randomization test) (S11B Fig). Structurally, the cortical areas contributing to contextual memory retrieval would form a network centered at areas 8Ad and LIP as anatomical hubs. The difference in location between functional and anatomical hubs suggests dynamic allocation of the functional hub, apart from the anatomical hub, in response to the cognitive requirements of contextual memory retrieval. Importantly, the functional hub (area 9/46d) corresponds to the lesion-effective site, damage of which induced task-specific impairment [18], whereas the anatomical hub (area 8Ad) does not.

### Dynamic Reallocation of the Functional Hub Depending on Task Demands

**Fig 4:**
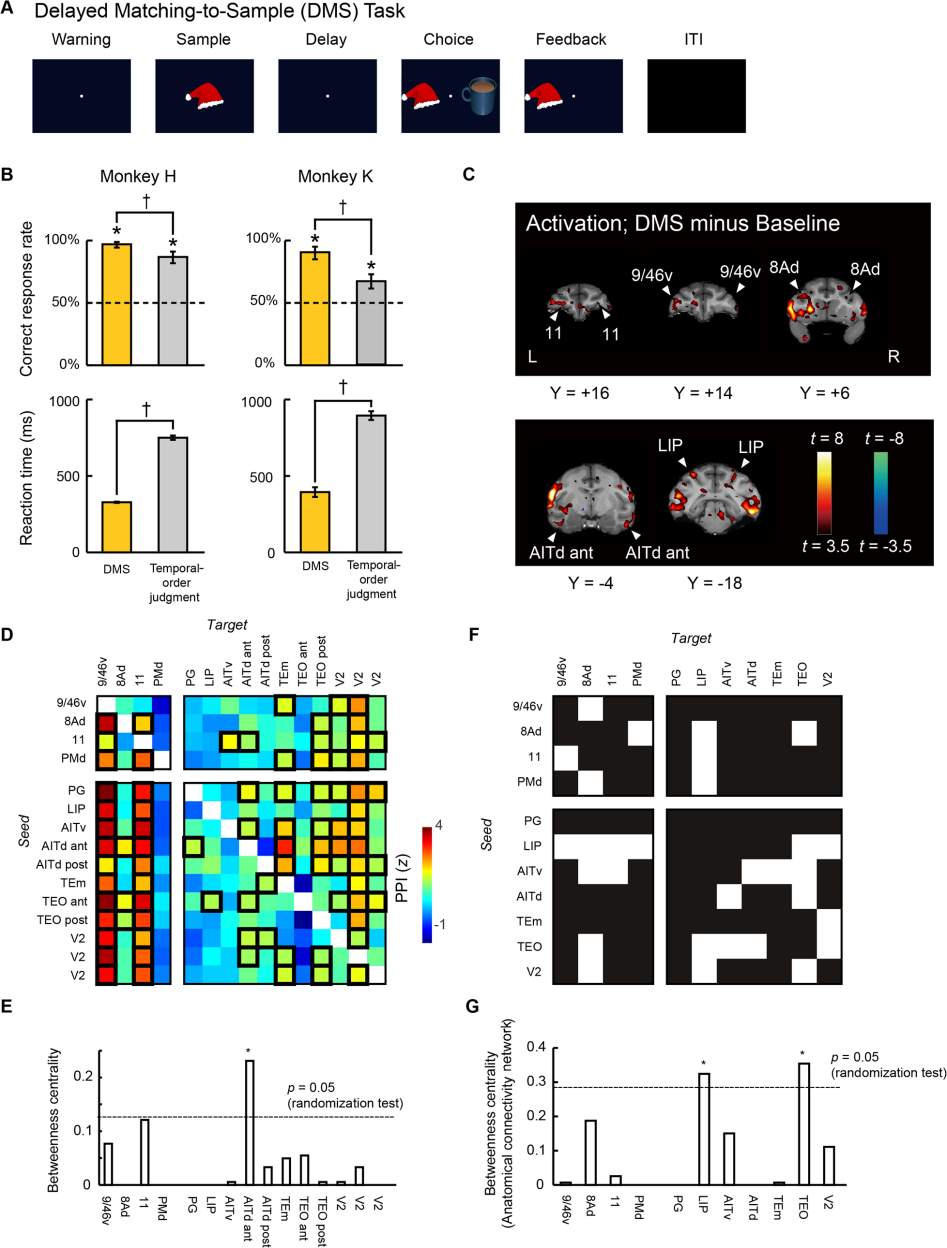
Dynamic reallocation of functional hub in response to demands of the delayed matching-to-sample task. (A) Trial structure in the DMS task. The monkeys were required to select the stimulus that had been presented as a sample stimulus. The stimuli of natural and artificial objects were chosen from Microsoft Clip Art or HEMERA Photo-Object database. The images provided in this figure are representations only and were not used in the experiment. (B) Percentages of correct responses (upper) and reaction times (lower) for each monkey. Behavioral performance of all the trials in the DMS task (yellow) is compared with that in the temporal-order judgment task (gray). The dashed line indicates the chance level. Error bars indicate SD across sessions. * *p* < 0.0003, † *p* < 0.002, *t*-test. (C) Brain regions active for the DMS task. Activation map is superimposed on coronal sections. (D) PPI matrix among the 15 homotopic areas identified in the DMS task. Rows and columns indicate seed and target regions, respectively. Significant connectivities are enclosed by thick black lines (*p* < 0.05 [FDR correction]). (E) Betweenness centralities of each area calculated based on (D). The dashed line indicates the significance at *p* < 0.05 (randomization test). * *p* < 0.05. (F) Anatomical connectivity matrix among the homotopic areas. Rows and columns indicate seed and target areas, respectively. A white (black) square indicates the presence (absence) of anatomical connection from row to column. Anatomical information is based on CoCoMac database. The areas to which the same labels are given in CoCoMac database area merged respectively. (G) Betweenness centralities of each area calculated based on (F). The dashed line indicates significance at *p* = 0.05 (randomization test). * *p* < 0.05.

To directly test if the location of the functional hub changes depending on demands for specific tasks, we conducted additional fMRI experiments in the same monkeys using a simple context-free memory task (delayed matching-to-sample [DMS] task) (Fig 4A; see Materials and Methods). More accurate and prompt responses during the DMS task in comparison with during the temporal-order judgment task (all *p* < 0.002, *t*-test) (Fig 4B) suggest qualitative differences in cognitive demands between the two tasks. Comparison of fMRI signals in the DMS task and baseline revealed significant activation of multiple cortical areas (Fig 4C, S4 Table). In the frontal cortex, significant bilateral activations were found in the ventral surface and ventral bank of the principal sulcus (area 9/46v), in the periarcuate area (area 8Ad), and in the orbitofrontal region (area 11). In the temporal cortex, the multiple bilateral activations were found in the anterior inferior temporal region (areas AITd and AITv). In total, we identified 15 homotopic areas with bilateral significant activation in both monkeys (see Materials and Methods).

On the basis of PPI patterns (Fig 4D and 4E) and axonal projection patterns (Fig 4F and 4G) among the 15 homotopic areas, we calculated betweenness centrality of each area (Fig 4E and 4G), similarly to the analyses for the temporal-order judgment task. The results showed that the location of the functional hub based on PPI patterns (area AITd: betweenness centrality, *p* = 0.001, randomization test) and the location of the anatomical hub based on axonal projection patterns (areas TEO and LIP: betweenness centrality, all *p* < 0.03, randomization test) were different. More importantly, the location of the functional hub in this context-free memory network was quite different from the location of the functional hub in the network of contextual memory (areas 9/46d and TEa). The location of the functional hub in the context-free memory network corresponded to the lesion-effective site for a similar task [49–52], as is the case with the contextual memory network—the location of the functional hub corresponds to the lesion-effective site for a temporal-order judgment task [18]. Therefore, the difference in location between the functional and anatomical hubs is suggested to be attributed to dynamic allocation of the functional hub.

### Prediction of Behavioral Performance and Behavioral Impairment after Lesioning

**Fig 5:**
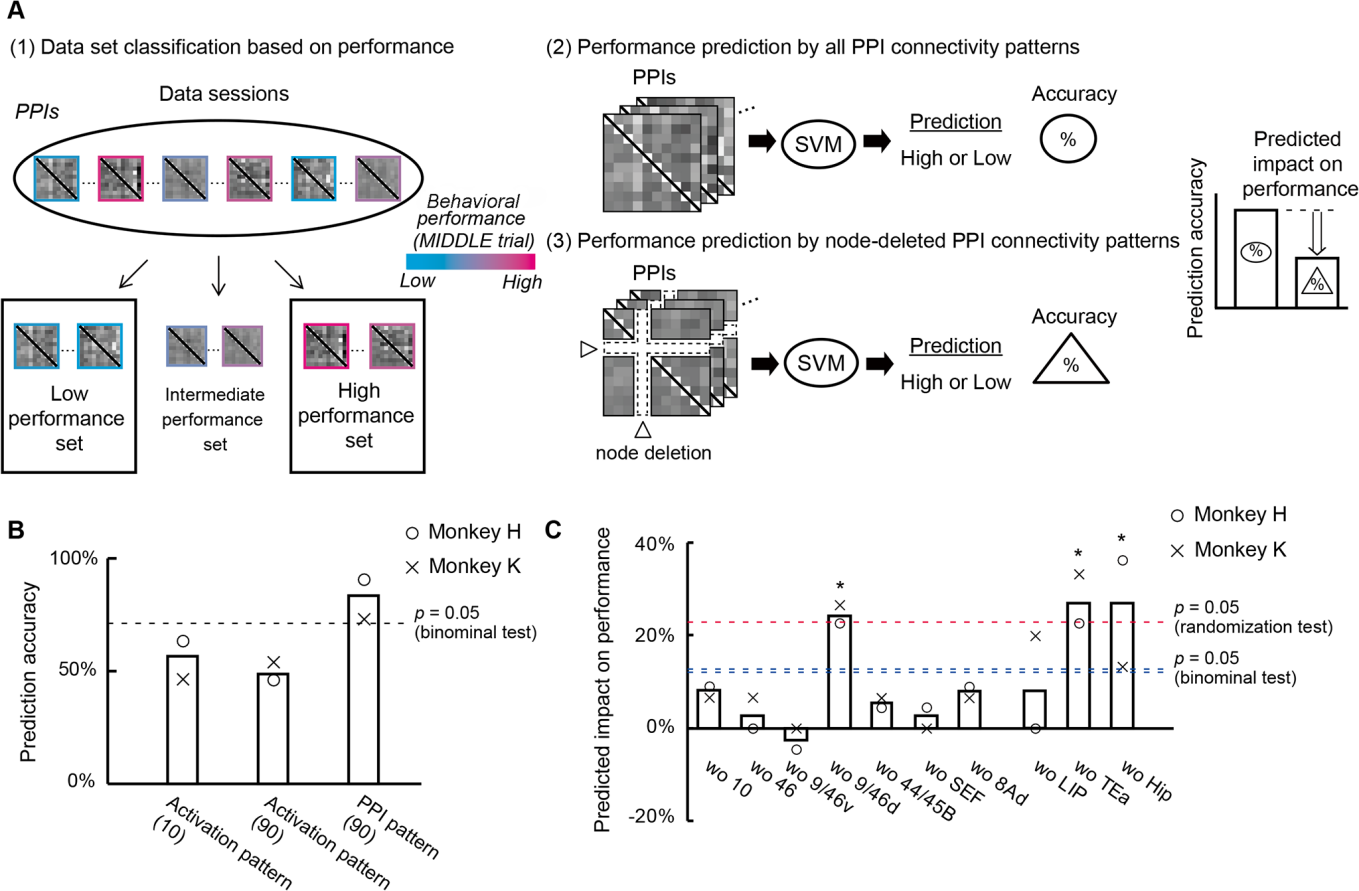
Prediction of behavioral performance and behavioral impairment after lesioning based on connectivity pattern. (A) Schematic of SVM-based MVPA prediction. (1) First, the whole data session set was divided into three classes based on behavioral performance in the MIDDLE trials (high, intermediate, and low performance sets). (2) Second, performance levels (high or low) in each session were predicted from the PPI connectivity pattern. (3) Finally, using node-deleted PPI connectivity patterns, SVM prediction was conducted. By comparing the prediction accuracies between (2) and (3), the predicted impact on performance after removal of a certain area was assessed. (B) Accuracy of behavioral performance prediction using activation patterns among the ten areas with ten features (left), activation patterns with 90 features (middle), and PPI connectivity patterns (right). The dashed line indicates accuracy significantly higher than chance (*p* = 0.05, binominal test, for group). Each circle and cross represents data for monkey H and monkey K, respectively. The number of features is shown in the brackets. (C) Predicted impact on performance after removal of the indicated areas is plotted. The red dashed line indicates significant predicted impact on performance (*p* = 0.05, randomization test, for group). * *p* < 0.05. The blue double dashed lines indicate prediction accuracy significantly higher than chance (*p* = 0.05, binominal test, for group). Bars below the blue double dashed lines indicate prediction accuracies still significantly better than chance after removal of the area.

We then examined whether the pattern of PPI connectivity among the ten homotopic areas encodes behavioral performance during the temporal-order judgment. We classified session-by-session PPI connectivity patterns into high or low behavioral performance sets for MIDDLE trials by using multivariate pattern analysis (MVPA) based on support vector machine (SVM) using nonlinear radial basis function kernels (Fig 5A, (1), (2); see Materials and Methods; for SVM using linear kernels, see S12 Fig). SVM prediction accuracy using all the PPI connectivity patterns among the ten homotopic areas was significantly higher than chance (monkey H: 90.9%, *p* = 1.1 × 10^−5^; monkey K: 73.3%, *p* = 0.03, binominal test) (Fig 5B, right), but activation patterns among the ten homotopic areas did not predict the performance class (all *p* > 0.1, binominal test) (Fig 5B, left). Even after matching the number of features applied to SVM for activation patterns to that for the PPI patterns, the prediction based on activation patterns did not give significantly high accuracy (all *p* > 0.5, binominal test) (Fig 5B, middle).

To simulate the effects of a cortical lesion on ability for contextual memory retrieval, we examined the effect of node deletion on prediction accuracy for behavioral performance. Using node-deleted PPI connectivity patterns (Fig 5A, (3)), SVM prediction accuracy was calculated and compared with that obtained by using the original connectivity patterns, and we estimated the impact on the ability to perform the task as a reduction in prediction accuracy (“predicted impact on performance”; see Materials and Methods) (Fig 5A(3) and 5C). A two-way ANOVA on the predicted impact on performance after removal of each area (monkey × removed area) demonstrated a significant main effect of removed area (*F*(9, 9) = 3.51, *p* = 0.03) without a significant main effect of monkey (*F*(1, 9) = 0.03, *p* = 0.85). Among the PFC areas, a significant predicted impact on performance was observed only after removal of area 9/46d (*p* = 0.03, randomization test [comparison with the distribution of randomly-edge-deleted network]; see Materials and Methods). On the other hand, removal of area 8Ad did not cause a significant reduction in prediction accuracy (*p* = 0.59, randomization test). These results indicate that the areal dissociation of lesion-induced memory impairment [18] was predicted by the node-deleted PPI connectivity pattern (see Discussion for the effect of removing a single connection). Outside the PFC, a significant predicted impact on performance was observed after removal of area TEa or the hippocampus (both *p* = 0.01, randomization test) (see S13 Fig for the differential contribution of outward and inward connectivity to the predicted impact on performance among areas 9/46d, TEa and the hippocampus).

**Fig 6:**
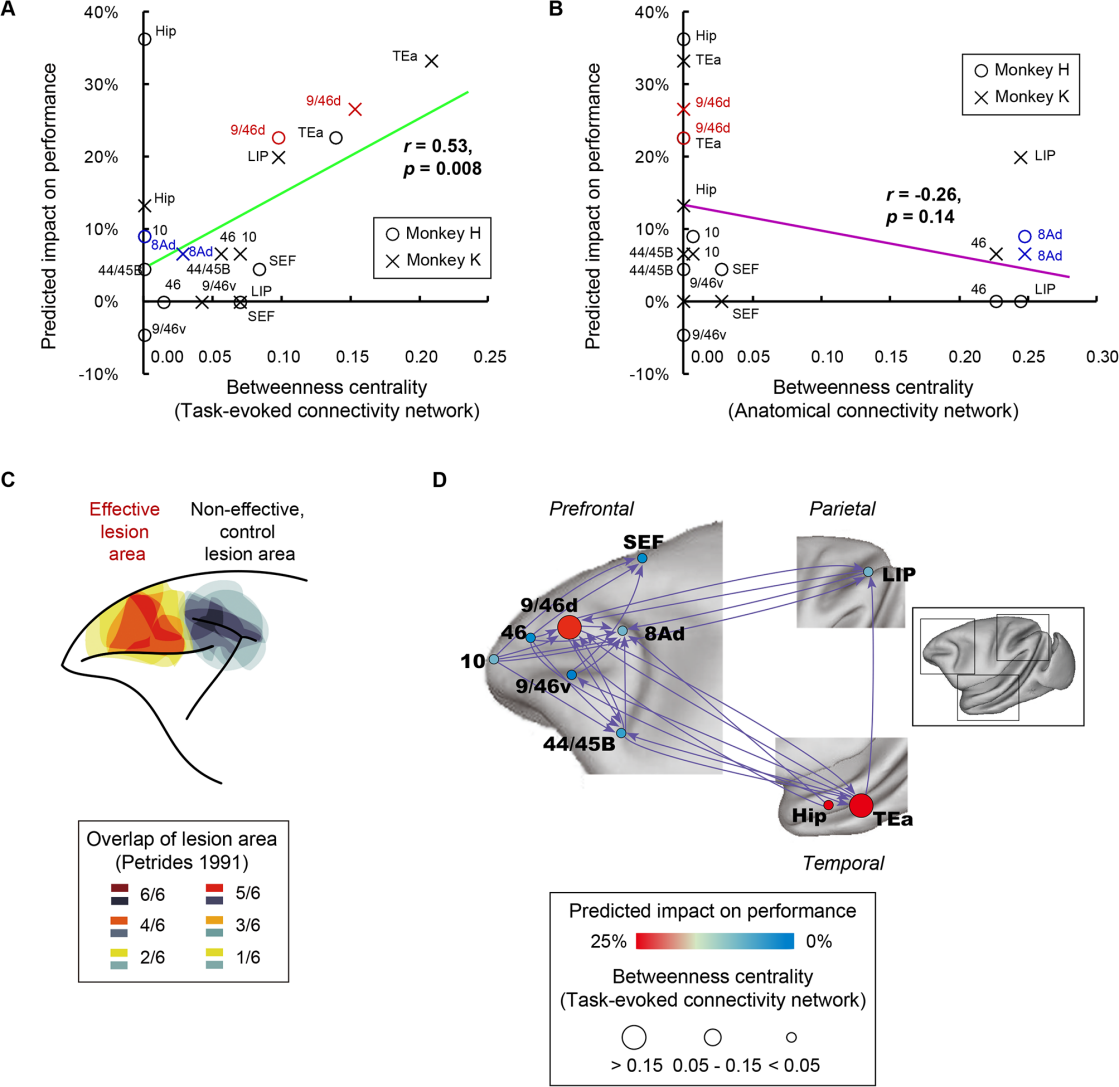
Relationship of betweenness centrality and predicted behavioral impairment after lesioning. (A) Betweenness centrality calculated based on task-evoked connectivity (horizontal axis) and predicted impact on performance (vertical axis) for each area for each monkey are plotted as a scattergram. The green line was fitted (*r* = 0.53, *p* = 0.008). (B) Betweenness centrality calculated based on anatomical connectivity (horizontal axis) and predicted impact on performance (vertical axis) for each area for each monkey are plotted as a scattergram. The purple line was fitted (*r* = -0.26, *p* = 0.14). (C) Areas where lesions induced impairment in temporal-order judgment; data were compiled from Petrides (1991) [18]. Red-yellow color code and gray code indicate the overlap of the lesion area among six hemispheres for mid-dorsolateral prefrontal area (effective lesion area) and periarcuate area (noneffective, control lesion area), respectively. (D) Schematic illustration of interareal connections. PPIs with *p* < 0.01 (FDR correction) are displayed as directed edges for display purpose. Node color indicates predicted impact on performance. Node diameter represents betweenness centrality. Note that causality cannot be inferred from PPI directionality.

We compared the predicted impact on performance after lesioning (Fig 5C) and the betweenness centrality based on task-evoked connectivity network (Fig 3E) for each area in each monkey. An analysis of covariance (ANCOVA) on predicted impact on performance after removal of each area (monkey × betweenness centrality) revealed a significant main effect of betweenness centrality of the removed area (*F*(1, 16) = 6.65, *p* = 0.02), but no significant main effect of monkey (*F*(1, 16) = 0.22, *p* = 0.64) or interaction between monkey and betweenness centrality (*F*(1, 16) = 0.85, *p* = 0.36). Moreover, we found a significant positive correlation between the betweenness centrality and the predicted impact on performance (*r* = 0.53, *p* = 0.008) (Fig 6A). These observations indicate that removal of an area with higher betweenness centrality in the task-evoked connectivity network causes a larger reduction in prediction accuracy. Contrarily, no significant correlation was observed between the betweenness centrality based on anatomical connectivity network (Fig 3I) and the predicted impact on performance (*r* = -0.26, *p* = 0.14) (Fig 6B). Even in a network containing the larger set of areas that included nonhomotopic areas (total of 39 areas; see Table 1), the predicted impact on performance correlated more highly with betweenness centrality based on task-evoked connectivity than with betweenness centrality based on anatomical connectivity (*p* = 8.8 × 10^−17^, paired *t*-test) (S14 Fig; see “Prediction with the Network with a Larger Set of Areas” in S1 Text). These observations suggest that severity of behavioral impairment induced by a focal lesion is predicted from the task-evoked connectivity network, but not from the anatomical connectivity network.

## Discussion

In the present study, we identified the macaque cortical network for temporal-order retrieval and revealed that area 9/46d acted as a hub in the task-evoked connectivity network, but not in the anatomical connectivity network. The task-specific functional hub in this dynamic task-evoked network accurately corresponded to the well-documented lesion-effective site, avoiding the neighboring non-lesion-effective site [18] (i.e., severe temporal-order memory impairment after lesioning in area 9/46d, but not in area 8Ad; Fig 6C). We then quantitatively estimated the behavioral impact of lesioning in each activated area. We found that predicted severity of impairment was proportional to the network hubness (betweenness centrality) of the virtually lesioned area in the task-evoked connectivity network rather than in the anatomical network.

This study is the first demonstration, to our knowledge, of the whole-brain mapping for cognitive control functions on contextual memory in macaque monkeys. In human neuroimaging studies, widespread regions in the PFC are reported to contribute to temporal-order retrieval with comparison between temporal-order memory and item recognition memory [19,20]. With more stringent comparisons between different demand levels of temporal-order retrieval, several PFC areas were consistently responsive to cognitive control of memory [21]. In the present study, even with applications of this stringent comparison, we identified multiple macaque PFC areas active for contextual memory retrieval, similarly as in humans.

The PPI measure was used to evaluate the network architecture of the task-based fMRI data. PPI measure is a change in functional connectivity modulated by task demand (i.e., difference in connectivity between the different conditions in the task). Thus, the PPI network is qualitatively different from static networks determined by anatomical connections or DTI, and the network connectivity patterns should dynamically change across different tasks. In addition, there is no simple correspondence between outward/inward connectivities and anterograde/retrograde anatomical connections. This is because task-evoked connectivity reflects both direct and indirect anatomical connections, and it is not fully understood how anatomical connections contribute to task-evoked connectivity [37,42].

Theoretically, in the PPI, the directional contribution in the PPI is established in the relationship between seed and target areas, whereas causality cannot be inferred from the PPI [37]. In the present study, owing to directionality of the PPI, estimation of the hierarchical structure identified the monkey area 10 to be situated at the highest level in the hierarchical structure among the PFC areas (S4 Fig), consistent with the proposed function of the human frontal pole [6,53,54]. We also conducted additional network analyses without any assumptions of directionality of PPI. The analyses showed that area 9/46d was a hub and area 8Ad was not (Fig 3F and 3G, S9 Fig), similarly to the results of the original analysis (Fig 3D and 3E). Therefore, the hub structure determined by PPI network is sufficiently robust not to be affected by the directionality of PPI.

We showed that lesions of a task-specific functional hub with high betweenness centrality (not of an anatomical hub) would bring severe behavioral effect. Our conclusion is corroborated by actual lesion studies. For the temporal-order judgment task, the functional hub area in the present study (area 9/46d) corresponds to the lesion-effective site in a previous controlled study [18], while the anatomical hub area (area 8Ad) corresponds to the lesion-ineffective site in the previous study (Fig 6C) [18]. For a simple memory task (DMS task) (Fig 4), the functional hub area in the present study (area AITd) corresponds to the lesion-effective site in previous studies [49–52], while the anatomical hub area (area TEO) corresponds to the lesion-ineffective site in the previous studies [49–52].

Several studies have computationally inferred the effects of lesions by evaluating simulated changes of network measures within structural networks [10,55] or within simulated functional connectivity networks [56,57]. These earlier computational studies did not commit specific cognitive functions nor did they predict behavioral impairment. In the present study, the analysis of a task-evoked connectivity network through the use of SVM enabled us to estimate the behavioral impact of lesioning by measuring the reduction in prediction accuracy caused by removing specific areas and connections. The validity of the current approach is demonstrated through the consistency of our results with those of an earlier controlled lesion study in monkeys [18]. That is, area 9/46d, deletion of which results in a significant decrease in the classification performance of SVM, corresponds to a lesion-effective site in the previous study, while area 8Ad, deletion of which results in no change in the classification performance, corresponds to a lesion-ineffective site in the previous study (Fig 6C and 6D). Incidentally, for the DMS task, we could not conduct the classification analysis, because the task was easy for monkeys and the rate of correct response, which is to be classified with SVM, was nearly 100% consistently across all experimental sessions.

We focused on activity and connectivity in MIDDLE trials in which there is a higher cognitive demand for temporal-order judgment than in BOTH-END trials. Therefore, evaluation of the predictability for session-by-session performance of MIDDLE trials (not of BOTH-END trials) is appropriate in the SVM analysis. The higher predictability of behavioral performance achieved by interareal task-evoked connectivity, compared with that achieved by areal activations (Fig 5B), suggests that the primary determinant of a deficit is attributable, not simply to specific cognitive processing within each of these PFC areas, but to intercommunicated signal flow patterns in a highly ordered PFC network for contextual memory retrieval (Figs 3E and 6D).

The hub regions tend to have more connections than other regions. We also compared the amount of decrease in classification accuracy after removing a single connection, or a single edge, in the PPI network. The results showed that removing a single connection from a hub region had a larger effect than removing a single connection from a nonhub region (hub region: 2.33 ± 0.63%; nonhub region: 1.45 ± 0.30%), although differences were marginally significant (hub region versus nonhub region: *p* = 0.058, *t*-test). Much smaller effects were obtained after removing a single connection than after removing all the connections to/from one region, as expected. The results suggest that our classification analysis is robust enough so that the prediction accuracy was not largely changed after removing only a single connection.

It is known that lesions of a single node may often result in changes in the connectivity of the remaining network nodes. In the present study, however, despite the strong assumption that the connectivity in the remaining network does not change after lesions, our classification methods successfully predicted the behavioral effect after removal of area 9/46d, of which even chronic lesion is known to result in severe impairment in monkeys [18]. Our results showed that even if the network connectivity among the remaining nodes changed, these indirect effects would be minor at least in the case of the contextual memory retrieval network.

Both betweenness centrality based on task-evoked connectivity and predicted impact on performance were highest in area 9/46d among the PFC areas (Figs 3 and 5), and these two measures showed a significant positive correlation among the task-related areas (Fig 6A), despite being mutually independent measures. These results converge to suggest that the severe task impairment induced by lesioning of area 9/46d reflects the functional “hubness” of this region. Thus, the vulnerable locus is suggested to correspond to the task-specific functional hub. Contrarily, we identified areas 8Ad and LIP, but not area 9/46d, as anatomical hubs with significant betweenness centralities both in the axonal projection-based anatomical network with the ten task-activated homotopic areas (Fig 3H and 3I) and in the network containing a larger set of areas (S11 Fig). These results of the anatomical hubs are largely consistent with the previous reports on the anatomical hubs in the whole-brain network of macaques [47,48]. The distribution of hubs was different between anatomical and task-evoked networks, and, unlike functional hubness, anatomical hubness did not predict the task impairment (Fig 6B). The different distribution of the anatomical and functional hubs suggests dynamic reallocation of the lesion-effective site, which corresponds to the functional hub, depending on the cognitive processes.

Clinically, as often seen in stroke human patients, slight locational differences between brain lesions affect severity of their behavioral aftereffects [7]. Our findings of the predicted severity of impairment will be a foundation of prediction of behavioral and cognitive impacts of damage or surgical intervention in human brains.

The present study proposes a possible causal link between task-specific cognitive impairment and lesions in the functional hub rather than those in the static anatomical hub. Further investigation on this hub-centric property of task-evoked networks involved in other cognitive functions, together with that on the behavioral impairment due to focal lesioning of the functional hub, will test the generalizability of our findings and establish a new principle on how a cortical area obtains its integrative role in the brain network function.

## Materials and Methods

All the experimental protocols, animal welfare, and steps for ameliorating sufferings were in full compliance with the Guidelines for Proper Conduct of Animal Experiments by the Science Council of Japan, with the University of Tokyo’s “Guidelines Regarding Animal Research and Animal-Experimentation Manual,” and with the “NIH Guidelines for the Care and Use of Laboratory Animals,” as well as with the Weatherall report, “The Use of Non-human Primates in Research.” The experimental protocol was approved by the University of Tokyo School of Medicine Animal Care and Use Committee (Permission Number, MED: P11-098).

### Subjects

We used two adult male macaque monkeys (*M*. *mulatta*: monkey H, 5 kg; monkey K, 7 kg) in the experiments. Monkeys were housed in standard primate cages in an air-conditioned room under 12/12-h light-dark cycle. Toys and puzzle feeders were provided for environmental enrichment. Monkeys were given primate food (Oriental Yeast) supplemented with fruits and vegetables. Detailed procedures for fMRI were previously described [34] and will be described here only briefly. Before fMRI scanning, an MRI-compatible version of a ring-type head fixation device [34,58] was attached to the monkeys. Surgery for attachment of the head-fixation device was conducted in aseptic conditions under general anesthesia with sodium pentobarbital (5 mg/kg/h, IV) and xylazine (2 mg/kg, IM), supplemented as needed. Monkeys were given postsurgical analgesics (ketoprofen, 1 mg/kg/d, IM) for at least 3 d and postsurgical prophylactic antibiotics (benzylpenicillin, 20,000 unit/kg/d; ampicillin, 100 mg/kg/d, IM; or enrofloxacin, 5mg/kg/d, subcutaneous injection) for 2 wk as described previously [59,60]. All monkeys used in the present study are alive and healthy and are currently engaged in experiments for other studies.

### Behavioral Procedures for Temporal-Order Judgment

The monkeys performed a temporal-order judgment task modified for fMRI (Fig 1A). Online behavioral control and reward delivery were controlled using custom-made PC software [28]. In a custom-made MRI-compatible monkey chair (Nakazawa, Tokyo, Japan), each monkey manipulated an optical fiber-based, custom-made three-way joystick with one of its forelimbs. An optical fiber-based photoelectric sensor (Omron, Tokyo, Japan) was used to monitor the movements of each of the other three limbs. Eye position was monitored at 120 Hz using an infrared-sensitive CCD camera (ISCAN, Massachusetts, United States of America).

Each trial began when the monkey pulled the joystick to trigger presentation of a fixation point (“Warning,” the duration varied between 6 s to 9 s on a trial-by-trial basis for monkey H and was 3 s for monkey K). A list of stimuli was then presented serially (“Cue,” the list consisted of six stimuli for monkey H and four for monkey K). Each stimulus was presented at the center of the monitor (for 1.5 s with monkey H and 1.1 s with monkey K) and was followed by an interstimulus interval (0.7 s for monkey H and 0.6 s for monkey K). The stimuli were selected in a pseudorandom order from a pool of 1,200 pictures of natural or artificial objects (Microsoft Clip Art or HEMERA Photo-Object database [Source Next, Tokyo]), which were cropped and presented to the monkeys at 3° × 3° in visual angles. Typically, each picture was presented only once (twice at most) in each session. The last stimulus was followed by a delay period (“Delay,” duration varied between 7.5 s and 8.5 s on a trial-by-trial basis for monkey H and was 5.5 s for monkey K). Finally, two stimuli from the studied list were simultaneously presented, one each on the right and left (“Choice”). If the monkey responded by moving the joystick in the direction of the stimulus that had been presented more recently, the monkey received juice drops accompanied by a distinctive secondary visual reinforcement (“Feedback”). A secondary visual reinforcement was used to support operant learning for the task in monkeys from the training phase. Incorrect choices resulted in termination of the trial without reward. Trials were separated by an intertrial interval, during which the screen was black. If any limbs moved during the trials, the optical sensors detected the movement, and the trial was aborted immediately. For monkey H, after a correct choice, two other stimuli in the same list were presented for judgment (see “Behavioral Effects due to the Inclusion of End Stimuli” in S1 Text).

In the judgment stage, two types of trials were performed: (1) trials in which the stimulus pair in Choice included neither the initial nor last end stimuli in the list (MIDDLE trial) and (2) trials in which at least one of the paired stimuli was either the initial or last end stimuli in the list (EITHER-END trial). Among the EITHER-END trials were trials in which the Choice pair consisted of the initial and last end stimuli in the list; this was termed a BOTH-END trial (Fig 1A).

### MRI Acquisition

Functional images were acquired in a 4.7-T MRI scanner (Biospec 47/40, Bruker, Ettlingen, Germany) with 100 mT/m actively shielded gradient coils and a transceiver saddle RF coil (Takashima, Tokyo, Japan) [34,59–64]. In each session, functional data were acquired using a gradient-echo echo-planar imaging (EPI) sequence (1-shot, TR = 2.5 s, TE = 20 ms, flip angle = 80°, 1.25 × 1.5 mm^2^ in-plane resolution, 64 × 96 matrix, slice thickness = 1.5 mm, interslice gap = 0.2 or 0.25 mm, 30 or 27 horizontal slices covering the whole brain). T2-weighted spin-echo (RARE) images with the same geometry as the EPI were also scanned. Each run consisted of 120 functional volumes (5 min; the first three volumes were discarded in the analysis), including typically two MIDDLE trials and two BOTH-END trials for monkey H and three MIDDLE trials and three BOTH-END trials for monkey K. In separate sessions, high-resolution T1-weighted structural images were scanned using a 3-D MDEFT sequence (0.5 mm isotropic). High-resolution EPI (32-shot, TR = 2.5 s, TE = 20 ms, flip angle = 80°, 0.625 × 0.75 mm^2^ in-plane resolution, 128 × 192 matrix, slice thickness = 0.8 mm with no gap, 62 horizontal slices covering the whole brain) was also acquired to serve as the template image for spatial normalization (see below).

### Identification of Regions Active for Temporal-Order Judgment

Data were analyzed using SPM5 (http://www.fil.ion.ucl.ac.uk/spm/). Functional images were corrected for slice timing, realigned, spatially normalized to the template image with interpolation to a 1 × 1 × 1 mm^3^ space, and smoothed with a Gaussian kernel (2.5 mm full width at half maximum [FWHM]). The template image was constructed from the high-resolution EPI of monkey H by coregistering it to monkey H’s anatomical MDEFT image arranged in bicommissural space with the origin at the anterior commissure [26,28,34,64].

We used SPM5 to conduct voxel-wise statistical analyses based on the general linear model (GLM). The analyses included the following predictors: the Cue stimulus onsets; the Choice onsets in the MIDDLE trials, BOTH-END trials, EITHER-END trials with Choice stimulus pair including only initial end stimulus, and EITHER-END trials with Choice stimulus pair including only last end stimulus; and the timing of errors. These events were modeled as delta functions convolved with a canonical hemodynamic response function and its temporal and dispersion derivatives. Six parameters of head motion derived from realignment were also included in the model as covariates of no interest. Data were high-pass filtered using a cutoff of 128 s. Data from 34 sessions for monkey H and 31 sessions for monkey K were analyzed. The analyses were restricted to the hits in the Choice periods. The numbers of MIDDLE, BOTH-END, and EITHER-END trials were 501, 693, and 1873, respectively, for monkey H, and 283, 389, and 965, respectively, for monkey K. The numbers of trials in each condition were sufficient enough to obtain reliable fMRI signals and to compare the difference across conditions. Misses were included in GLM analysis, but we did not compare the fMRI signals during miss trials. The numbers of misses in MIDDLE, BOTH-END, and EITHER-END conditions were 183, 1, and 193, respectively, for monkey H and 192, 94, and 436, respectively, for monkey K. The group analysis of these data was conducted using a random effect model by treating each session’s data from both monkeys equally as a random effect. The regions active during temporal-order judgment were identified by comparison of blood-oxygenation-level-dependent (BOLD) signals between the MIDDLE and BOTH-END conditions [21].

The coordinates of the activation peaks with a threshold of *p* < 0.05 with FWE correction were included in Table 1. To detect homotopically activated areas, we used the nearest-neighbor algorithm [28,65] as follows. We flipped the *x*-coordinates of the activation map in the left hemisphere and superimposed them on the right hemisphere. Then, each significant activation peak derived from the left hemisphere was paired with its nearest peak derived from the right hemisphere if the distance between them was less than 5 mm (areas 10, 46, 9/46d, 44/45B, 8Ad, and TEa). In addition, nearest neighbors for prefrontal peaks of area 9/46v and the supplementary eye field were detected among contralateral activations with *p* < 0.005 corrected by FDR [66]. Nearest-neighbor bilateral activations with *p* < 0.005 corrected by FDR were also detected in hippocampus and posterior parietal cortex (area LIP), the anatomical connections of which with the prefrontal cortex were suggested to be involved in prefrontal executive and memory control [4]. These ten bilaterally activated areas were termed homotopic areas and were also included in Table 1. The peaks were labeled by referring to the atlas of Petrides [4] and the atlas of Paxinos et al. [67]. The region of interest (ROI) for each peak was defined as the significant voxels (*p* < 0.001) within a 2-mm radius around the peak. For visualization purposes, statistical maps were projected onto a three-dimensional representation of the cortical surface of monkey H using Caret software (http://brainvis.wustl.edu/wiki/index.php/Caret:About) [68].

To confirm intersubject reproducibility of activated homotopic areas, we examined whether significant activations were located in individual monkeys within the ROIs defined in the group analysis. A single-voxel threshold of *p* < 0.05 corrected by FWE within each homotopic region was used for individual data analysis. *z*-values of significant peaks for each monkey are listed in S1 Table.

To test whether different reaction times in the MIDDLE and BOTH-END conditions could explain the activations detected above, in a separate analysis the parametric modulation method for coding reaction times into GLM was applied using three types of predictors for MIDDLE and BOTH-END trials [21]. The first predictor coded the main effect of MIDDLE and BOTH-END trials, the second predictor coded the reaction times in the MIDDLE and BOTH-END trials, and the third predictor coded the trial type (+1 for MIDDLE and -1 for BOTH-END trials). We then determined whether significant peaks were detected in this analysis (S2 Table). A single-voxel threshold of *p* < 0.05 corrected by FDR was used.

We also compared fMRI signals between MIDDLE and EITHER-END conditions. We then examined whether significant activations were detected in this comparison within the ROIs defined in the original analysis (MIDDLE minus BOTH-END) (S3 Table). A single-voxel threshold of *p* < 0.05 corrected by FWE within each homotopic region was used.

### Psychophysiological Interaction Analysis

PPI [69] was estimated among the homotopic areas using SPM5 to determine whether the temporal-order retrieval load affects the connectivity among the identified areas. The “physiological” time series extracted from a seed ROI was corrected for variance associated with parameters of no interest, deconvolved with the hemodynamic responses, multiplied by a parameter encoding the relevant “psychological” contrast (MIDDLE > BOTH-END), and reconvolved to form a “psychophysiological interaction” (“PPI”) predictor: these three predictors (“physiological” time series, “psychological” contrast, and “PPI”) were entered into a design matrix alongside the same nuisance predictors used for the GLM analysis described above. The effect size of the PPI at target ROIs was evaluated as the beta estimate for the “PPI” predictor averaged across all sessions for the two monkeys.

After calculating all the possible combinations of PPIs among the ten homotopic areas (10 × (10–1) intrahemispheric connectivities for both hemispheres, without autorecursive ones), we applied a three-way ANOVA (laterality [left or right] × seed area × target area). We then averaged PPIs in the left and right hemispheres of the two monkeys and calculated *z*-values. We also estimated interhemispheric PPI connectivities and calculated *z*-values.

The topology of the connectivity networks among the ten homotopic areas were examined by calculating network metrics. For each area (node), the network measure of “betweenness centrality” was computed using Brain Connectivity Toolbox (https://sites.google.com/site/bctnet/) [40]. The betweenness centrality is the fraction of shortest paths passing a given node. In the analysis, the distance from node *A* to node *B* (*d*_A->B_) was calculated as the inverse of the PPI *z*-value from node *A* to node *B* (*d*_A->B_ = 1/PPI_A->B_) [40] (see S2 Text for the code). If the PPI *z*-value was negative or zero, the distance was defined as infinity. To estimate the statistical significance of betweenness centrality, we generated randomized networks 100,000 times by changing the pattern of connectivity among the ten homotopic areas [70,71]. For each randomized network, we computed the betweenness centrality of each node, after which we estimated the distribution of betweenness centrality values by pooling all the values from all the nodes of all the randomized networks. We then compared the experimentally observed betweenness centrality values with the computed distribution (S7 Fig).

We also calculated network metrics from a binarized network [40,72] (thresholded at *p* < 0.05 with FDR correction). For each node, the following network measures were computed using Brain Connectivity Toolbox: “degree,” the total number of connections to a certain node, “indegree,” the number of inward connections, “outdegree,” the number of outward connections; the proportion of outdegree to degree; and “betweenness centrality,” the fraction of shortest paths passing through a certain node (see S2 Text for the code). To estimate the statistical significance of degree and betweenness centrality, we generated randomized networks 100,000 times while maintaining the total number of connections among the ten homotopic areas. For each randomized network, degree and betweenness centrality were computed for each node. We estimated the distribution by pooling all the values from all the nodes of all the randomized networks and then compared the experimentally observed degree and betweenness centrality values with the computed distributions.

We conducted network analyses without any assumptions of directionality in two ways: (1) an analysis using a network in which the weight of the connection between A and B is equal to the mean value of PPI_A->B_ and PPI_B->A_ and (2) an analysis using a network in which the weight of the connection between A and B is equal to the larger values of PPI_A->B_ and PPI_B->A_. We calculated the betweenness centrality of each node (see S2 Text for the code).

We also calculated the network measure of betweenness centrality in a network containing a larger set of areas, including nonhomotopic areas. In addition to the ten homotopic areas defined above, bilateral peaks in which at least one of the bilateral activations (MIDDLE > BOTH-END) exceeded a threshold of *p* < 0.05 with FWE correction and both bilateral activations exceeded a threshold of *p* < 0.05 with FDR correction were used for the analysis. In total, 39 areas, including 29 nonhomotopic areas, were employed in the further PPI network analysis.

### Prediction of Behavioral Performance Using Support Vector Machine

We used MVPA based on SVM [64,73–75] to determine whether the PPI connectivity pattern encodes behavioral performance. We conducted most of our analyses on a supercomputer platform (TSUBAME 2.5 cluster system in Global Scientific Information and Computing Center, Tokyo institute of Technology, Japan). This system implements 76,038 cores and provides 2,843 TFlop/s with Linpack performance. Before the analysis, the whole data session set was divided into three subsets based on performance in the MIDDLE trials in each session (high, intermediate and low performance sets) (Fig 5A, (1)). The sessions in which the performance in the MIDDLE trials (correct response rates) was in the top (or bottom) tertile of all the sessions were classified as the high (or low) performance set, and the rest were classified as the intermediate set. Atypical sessions, in which the correct response rate was less than 50%, were omitted. The PPI beta-values (MIDDLE > BOTH-END) for each connection in each session from the high and low performance set data were analyzed for each monkey (22 sessions for monkey H and 15 sessions for monkey K). SVM was used to discriminate between sessions belonging to two different classes (high or low performance set) using soft-margin separation (Fig 5A, (2)). SVM classifications used nonlinear radial basis functions (*f*(**x**,**y**) = exp(-*γ* |**x**-**y**|^2^), **x**, **y**: training vectors) implemented in LIBSVM (http://www.csie.ntu.edu.tw/~cjlin/libsvm/) [76]. SVM found the maximal margin while allowing some misclassification, and the relative importance of maximizing the margin versus minimizing the misclassification was controlled by the constant *C*. The MVPA was achieved by splitting the data into a training set and a test set. A standard *k*-fold cross validation testing regimen was used [64], where *k* was equal to the number of sessions. The data from each session were set aside and used, in turn, as the test data; the remaining data were used as the training data. This method generated *k* sets for the SVM training as well as test sets that were used to derive the overall classification accuracy from the proportion of correct (see S2 Text for the code). Two parameters, *C* and *γ*, in the SVM were chosen to optimize the prediction classification accuracy for each monkey. The statistical significance of the prediction accuracy was evaluated using the binominal test. We employed SVM using nonlinear radial basis functions because it classifies data, irrespective of its distribution, more robustly than SVM using linear kernels [76,77]. We also used SVM classifications with linear kernels to test if the results were reproduced. For classification accuracy using a network with a larger set of areas, we conducted a “feature selection” procedure before SVM (see “SVM Analysis Using a Larger Set of Areas” in S1 Text).

We also tested whether the activation pattern encodes behavioral performance by applying the SVM procedure to the activation beta-values (MIDDLE > BOTH-END) of each homotopic area in each session from the high and low performance sets. In a separate analysis, to match the number of features in activation patterns to that of the PPI patterns (i.e., 90), each homotopic area was partitioned into nine parts, and nine activation beta-values (MIDDLE > BOTH-END) of each homotopic area in each session were used for the SVM analysis.

To computationally examine whether removal of an area affected prediction accuracy, we conducted an SVM prediction analysis using the node-deleted PPI connectivity patterns (i.e., 18 PPI connectivities to/from a certain node were omitted from the PPI connectivity pattern among the ten homotopic areas). The prediction accuracy of the node-deleted PPI connectivity patterns for each node was estimated and compared with that using all the connectivity patterns. A reduction in prediction accuracy was defined as “predicted impact on performance” (Fig 5A, (3)) (see S2 Text for the code). The predicted impact on performance was calculated for each node for each monkey. A two-way ANOVA on the predicted impact on performance by removal of each area (monkey × removed area) was performed after arcsine transformation. To estimate the statistical significance of a predicted impact on performance, we generated connectivity patterns 100,000 times using 18 randomly deleted connectivities, and prediction accuracy was calculated for each pattern. The distribution of predicted impact on performance after arcsine transformation was estimated by fitting the normal distribution function. The statistical significance of the observed predicted impact on performance was evaluated by comparing the value with the distribution.

To examine the relationship between betweenness centrality and predicted impact on performance, we performed an ANCOVA on predicted impact on performance after removal of each area (monkey × betweenness centrality). Betweenness centrality and predicted impact on performance after removal of each area were calculated for each monkey and were used for the analysis.

For the areas that showed significant predicted impact on performance (areas 9/46d, TEa, and the hippocampus), the contributions of outward and inward connectivity were assessed. We compared the PPI beta-values of each connection from high and low performance sets and calculated *t*-values, which were then transformed to *z*-values that were termed classification indices. A three-way ANOVA on the classification indices for the connections to and from removed areas (monkey × area × directionality [outward or inward]) was then conducted.

### Anatomical Connectivity Network

The anatomical connectivity data set comprises the data from tract-tracing studies collated in the online neuroinformatics data base CoCoMac (http://cocomac.g-node.org) [41]. The connection data consisted of directed projections represented in binary format (connection present = 1, connection absent = 0). No distinction was made between strong versus weak connections or between connections that were shown to be absent versus those for which no data were available. The matrix contained no self-connections. The cortical connection matrix was constructed from a set of ten homotopic areas (Fig 3H) and also from a larger set of areas that included nonhomotopic areas (28 areas) (S11A Fig). For each node, the betweenness centrality was computed. To estimate the statistical significance of betweenness centrality, we generated randomized networks 100,000 times while maintaining the total number of connections. For each randomized network, betweenness centrality was computed for each node. We estimated the distribution by pooling all the values from all the nodes of all the randomized networks and then compared the observed betweenness centrality value with the computed distributions.

### Identification of Lesion Areas in a Previous Lesion Study

To compare the areas activated in the present study with areas lesioned in a previous study [18], the extents of lesioned areas in the MDL-PFC were manually drawn for six hemispheres in three monkeys based on the original figures in the previous study [18]. All the lesioned areas were superimposed on the atlas, and the extent of overlap was assessed. These procedures were also performed for a control lesion in the periarcuate area.

### Experiments with Delayed Matching-to-Sample Task

We conducted additional fMRI experiments in the same monkeys using a DMS task (Fig 4A). Each trial began when the monkeys pulled the joystick to trigger presentation of a fixation point (“Warning,” duration: 1.5 s). A stimulus was then presented (“Sample,” duration: 0.5 s). After a delay (“Delay,” duration: 1.5 s), two stimuli, one of which had been presented in the Sample period, were presented simultaneously (“Choice”). If the monkey responded by moving the joystick in the direction of the stimulus presented in the Sample period, the monkey received juice drops accompanied by a distinctive secondary visual reinforcement (“Feedback”). Trials were separated by an intertrial interval (“ITI”), during which the screen was black. Functional images were analyzed using SPM5 in the same manner as the experiment for temporal-order judgment. For the voxel-wise statistical analysis based on the GLM, the time period from Sample period to Choice period (“DMS task”) was included as a predictor. The events were modeled as box-car functions convolved with a canonical hemodynamic response function and its temporal and dispersion derivatives. Six parameters of head motion derived from realignment were also included in the model as covariates of no interest. Data were high-pass filtered using a cutoff of 128 s. Data from five sessions for monkey H and five sessions for monkey K were analyzed. The group analysis of these data was conducted using a fixed-effect model. The regions active during DMS task were identified by comparison of the fMRI signals in the DMS task and baseline.

The coordinates of the homotopic activated peaks were listed in S4 Table according to the following criterion: (1) one of the bilateral peaks exceeded a threshold of *p* < 0.05 with FWE correction and both bilateral activations exceeded a threshold of *p* < 0.001, and (2) significant activations were located in individual monkeys within the 2-mm radius around the peak determined by the group analysis (a single-voxel threshold of *p* < 0.05 corrected with FWE within the radius). On the basis of PPI patterns and axonal projection patterns among the 15 homotopic areas listed in S4 Table, we calculated betweenness centrality of each area, similarly as for the temporal-order judgment task.

## Supporting Information

S1 Data Excel spreadsheet containing the underlying numerical data for Figs 1, 3, and 4–6 and S1, S3–S6, and S8–S14 Figs. (XLSX)

S1 Fig Behavioral performance of monkeys. Behavioral performance of each monkey during scanning sessions. Trials in which the stimulus pair included initial (or last) end stimulus in the list are termed INITIAL-END (LAST-END) trials. Upper panels show percentages of correct responses for each monkey. The dashed line indicates the chance level. Lower panels show reaction times for each monkey. Error bars indicate SD across sessions. * *p* < 10^−4^, *t*-test. † *p* < 10^−4^, †† *p* < 10^−5^, paired *t*-test.
(TIF)

S2 Fig Brain regions active revealed by the contrast of MIDDLE minus EITHER-END. (A) Activity related to temporal-order judgment revealed by the contrast of MIDDLE minus EITHER-END. An activation map is superimposed on the inflated brain: top, lateral view; bottom, anterior view. (B–D) Activation map is superimposed on transverse sections (B), coronal sections (C), and sagittal sections (D).
(TIF)

S3 Fig Task-evoked connectivities from/to area 10 and area 9/46d for each monkey. Corresponding data of individual monkeys for the data in Fig 3B and 3C. Each circle and cross represents data for monkey H and monkey K, respectively. Two bar plots in each column show *z*-values for PPIs from area 10 (A) or area 9/46d (B) to other ipsilateral homotopic areas (gray) and PPIs from other homotopic areas to area 10 (A) or area 9/46d (B) (white).
(TIF)

S4 Fig Task-evoked connectivity during temporal-order judgment. (A) Outward selectivities are plotted for each area. Error bars indicate SD. * *p* < 0.05 (Bonferroni correction). Area 10 has significant bias toward outward connectivity, whereas area 8Ad has significant bias toward inward connectivity. (B) Frequency distribution for 15 optimal hierarchical orderings in the PFC network. The boxes are shaded according to the relative occurrence of an area at a particular level across all the computed hierarchies. See “Hierarchical Structure in the Prefrontal Cortical Network” and “Estimation of Hierarchical Structure in the Task-Evoked Connectivity Network” in S1 Text.
(TIF)

S5 Fig Interhemispheric task-evoked connectivity during temporal-order judgment. (A, B) Effects of interhemispheric PPI. Two bar plots in each column show *z*-values of interhemispheric PPIs from area 10 (A) or area 9/46d (B) to other homotopic areas (gray) and from other homotopic areas to area 10 (A) or area 9/46d (B) (white). Dashed lines indicate significance at *p* = 0.05 with FDR correction. * *p* < 0.05 with FDR correction. (C) Interhemispheric PPI matrix among the ten homotopic areas. Rows and columns indicate seed areas and target areas, respectively. Significant connectivities are enclosed by thick black lines (*p* < 0.05 with FDR correction). Intrahemispheric (see Fig 3D) and interhemispheric connectivity patterns were significantly correlated (*r* = 0.78, *p* = 1.1 × 10^−19^). (D) Outward selectivities are plotted for each area. Error bars indicate SD. * *p* < 0.05 with Bonferroni correction.
(TIF)

S6 Fig Task-evoked connectivity for each monkey. PPI matrix among the ten homotopic areas for each monkey (top, monkey H; bottom, monkey K; left, intrahemispheric connectivity; right, interhemispheric connectivity). Rows and columns indicate seed areas and target areas, respectively. A significant correlation was observed between two monkeys (*r* = 0.25, *p* = 0.004).
(TIF)

S7 Fig Randomization test for betweenness centrality. (A) Schematic illustration of randomization test procedure. (1) Randomly rewired connectivity patterns among the homotopic were generated 100,000 times by shuffling the original 90 connectivity values. (2) For each randomized network, we computed the betweenness centrality of each node. (3) The random distribution of betweenness centrality values were estimated by pooling all the values from all the nodes of all the randomized networks. (B) The positions of the actual measured betweenness centrality of each area in the computed distribution. Data of betweenness centrality from Fig 3E are shown.
(TIF)

S8 Fig Network metrics in the binarized network. (A) Degrees (overall numbers of connections) are plotted for each area: white, outdegree (number of outward directed connections); black, indegree (number of inward directed connections). The dashed line indicates significance at *p* = 0.05 (randomization test). * *p* < 0.05. (B) Proportions of outdegree are plotted for each area. (C) Betweenness centralities are plotted for each area. The dashed line indicates significance at *p* = 0.05 (randomization test). * *p* < 0.05.
(TIF)

S9 Fig Betweenness centralities in the nondirectional network. (A) PPI matrix among the homotopic areas without assumptions of directionality. The weight of the connection between A and B is evaluated as larger values of PPI_A->B_ and PPI_B->A_. See also Fig 3D and 3F. (B) Betweenness centralities calculated based on (A). The dashed line indicates significance at *p* = 0.05 (randomization test). * *p* < 0.05.
(TIF)

S10 Fig Task-evoked connectivity in a network containing a larger set of areas. (A) PPI matrix among a set of 39 areas. Rows and columns indicate seed and target areas, respectively. Significant connectivities are enclosed by thick black lines (*p* < 0.05 with FDR correction). For both activation patterns and PPI connectivity patterns of the larger set, there was a significant correlation between two monkeys (for activation pattern, *r* = 0.26, *p* = 0.02; for PPI connectivity pattern, *r* = 0.15, *p* = 2.5 × 10^−17^). (B) Betweenness centralities are plotted for each area. The dashed line indicates significance at *p* = 0.05 (randomization test). * *p* < 0.05.
(TIF)

S11 Fig Anatomical connectivity network among a larger set of areas. (A) Anatomical connectivity matrix among a larger set of areas. Rows and columns indicate seed and target areas, respectively. A white (black) square indicates the presence (absence) of anatomical connection from row to column. Anatomical information is based on the CoCoMac database [41,47,48]. The areas to which the same labels are given in CoCoMac database are merged respectively. The projections to/from areas 8Ad, SEF, PMd, PMv, 24c, LIP, 5, 23b, TEpd, TEO, and TFO listed in the matrix are categorized as those to/from areas 8A, 6DR, 6DC, 6VR, 24, POa, PE, 23, CITd, PIT, and TF in CoCoMac, respectively. (B) Betweenness centralities calculated based on (A). The dashed line indicates significance at *p* = 0.05 (randomization test). * *p* < 0.05.
(TIF)

S12 Fig Prediction of behavioral performance and behavioral impairment with SVM analysis using linear kernels. (A) Accuracy of behavioral performance prediction using activation patterns among the ten areas with ten features (left) and PPI connectivity patterns (right) with SVM analysis using linear kernels. The dashed line indicates accuracy significantly higher than chance (*p* = 0.05, binominal test, for group). Each circle and cross represents data for monkey H and monkey K, respectively. (B) Betweenness centrality calculated based on task-evoked connectivity (horizontal axis) and predicted impact on performance (vertical axis) estimated using linear kernels for each area for each monkey are plotted as a scattergram. The black line was fitted (*r* = 0.46, *p* = 0.02).
(TIF)

S13 Fig Classification indices for areas 9/46d, TEa, hippocampus, and 8Ad. For areas 9/46d, TEa, and hippocampus, which showed significant predicted impact on performance, we compared the PPI values to and from deleted areas and estimated the contribution of inward and outward connectivity to the predicted impact on performance (“classification index”; see Materials and Methods). A three-way ANOVA (monkey × area [9/46d, TEa, hippocampus] × directionality [outward or inward]) of classification indices revealed no significant main effects of monkey, area, or directionality (all *p* > 0.2) or significant interactions between monkey and area or between monkey and directionality (all *p* > 0.3), though significant interaction between area and directionality was found (*F*(2, 96) = 5.00, *p* = 0.008). For area 9/46d, classification indices of outward directionality were significantly higher than those of inward directionality (*p* = 0.006, paired *t*-test) and were also significantly higher than the classification indices of other remaining connections (*p* = 0.008, *t*-test). For area TEa, classification indices of inward directionality were significantly higher than those of outward directionality (*p* = 0.04, paired *t*-test) and were also significantly higher than those of other remaining connections (*p* = 0.03, *t*-test). For hippocampus, there is a tendency for classification indices of outward directionality to be higher than those of inward directionality (*p* = 0.12, paired *t*-test). For comparison, in area 8Ad, there was no significant difference between the classification indices of outward and inward directionality and no significant increase of the classification indices of outward or inward directionality from those of other remaining connections (all *p* > 0.1). * *p* < 0.05, *t*-test. † *p* < 0.05, paired *t*-test.
(TIF)

S14 Fig Prediction of behavioral performance and behavioral impairment after lesioning based on connectivity pattern in the network with a larger set of areas. (A) Accuracy of behavioral performance prediction using activation patterns. The dashed line indicates accuracy significantly higher than chance (*p* = 0.05, binominal test, for group). The circle and the cross represents data for monkey H and monkey K, respectively. (B) Mean accuracy of behavioral performance prediction using PPI connectivity patterns after a “feature selection” procedure was plotted for the three ranges of number of selected features: 501 to 600 (left), 601 to 700 (middle), and 701 to 800 (right). The double dashed lines indicate accuracy for significantly higher than chance for monkey H (*p* = 0.05, binominal test). (C) Stability of correlation coefficient between predicted impact on performance and betweenness centrality as a function of the number of selected features. Betweenness centrality of each area was calculated based on task-evoked connectivity (green) and on anatomical connectivity (purple), respectively. (D) Betweenness centrality calculated based on task-evoked connectivity (horizontal axis) and predicted impact on performance (vertical axis) for each area for each monkey from the data point in (C) are plotted as a scattergram. The green line was fitted (*r* = 0.23, *p* = 0.02). (E) Betweenness centrality calculated based on anatomical connectivity (horizontal axis) and predicted impact on performance (vertical axis) for each area for each monkey from the data point in (C) are plotted as a scattergram. (F) Mean correlation coefficients between predicted impact on performance and betweenness centrality for the range of the number of selected features shown in (C). Betweenness centrality of each area was calculated based on task-evoked connectivity (green) and on anatomical connectivity (purple), respectively. A dashed line indicates a correlation coefficient of *r* = 0.19 (*p* = 0.05). † *p* < 10^−16^, paired *t*-test. Error bar indicates SD.
(TIF)

S1 Table Individual activation in homotopic areas. *Z*-values of significant peaks for each monkey at a voxel level of *p* < 0.05 corrected by FWE within group homotopic regions. † Significant only at a voxel level of *p* < 0.05 corrected by FDR within group homotopic regions. ‡ Significant only at a voxel level of *p* < 0.05.
(DOCX)

S2 Table Reaction time-corrected activation in homotopic areas. Significant peaks at a voxel level of *p* < 0.05 corrected by FDR. Coordinates are listed in monkey bicommissural space [26,28,34].
(DOCX)

S3 Table Activation revealed by the contrast of MIDDLE minus EITHER-END in homotopic areas. Significant peaks at a voxel level of *p* < 0.05 corrected by FWE within group homotopic regions. Coordinates are listed in monkey bicommissural space [26,28,34].
(DOCX)

S4 Table Activations in homotopic areas in delayed matching-to-sample task. Significant peaks at a voxel level of *p* < 0.05 corrected by FWE. Coordinates are listed in monkey bicommissural space [26,28,34]. † Significant only at a voxel level of *p* < 0.001.
(DOCX)

S1 Text Supplementary results and supplementary methods. (DOCX)

S2 Text Matlab-style codes for network analyses and SVM analyses. (DOCX)

## Abbreviations:

ANCOVA: analysis of covariance
ANOVA: analysis of variance
BOLD: blood-oxygenation-level dependent
DMS: delayed matching to sample
EPI: echo-planar imaging
FDR: false discovery rate
fMRI: functional MRI
FWE: family-wise error
FWHM: full width at half maximum
GLM: general linear model
ITI: intertrial interval
LIP: lateral intraparietal area
MDL: mid-dorsolateral
MRI: magnetic resonance imaging
MVPA: multivariate pattern analysis
PFC: prefrontal cortex
PPI: psychophysiological interaction
ROI: region of interest
SD: standard deviation
SEF: supplementary eye field
SVM: support vector machine
