# Supplementary figures and images for: Dynamically Allocated Hub in Task-Evoked Network Predicts the Vulnerable Prefrontal Locus for Contextual Memory Retrieval in Macaques
Source: PLoS Biol. 2015 Jun 30;13(6):e1002177. doi: 10.1371/journal.pbio.1002177 (PMC4488377; doi:10.1371/journal.pbio.1002177)

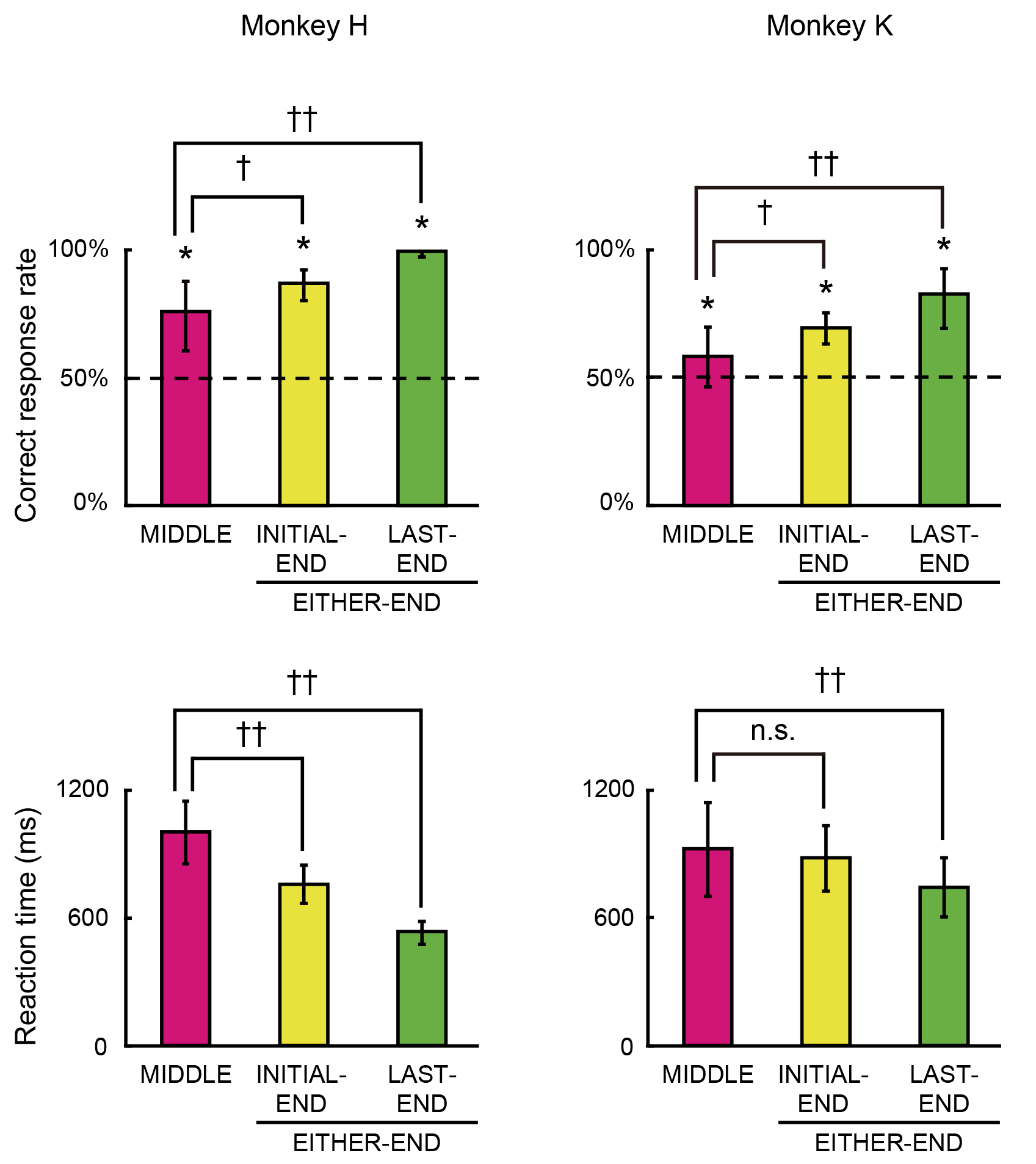

Supplement: S1 Fig — Behavioral performance of each monkey during scanning sessions. Trials in which the stimulus pair included initial (or last) end stimulus in the list are termed INITIAL-END (LAST-END) trials. Upper panels show percentages of correct responses for each monkey. The dashed line indicates the chance level. Lower panels show reaction times for each monkey. Error bars indicate SD across sessions. * p < 10−4, t-test. † p < 10−4, †† p < 10−5, paired t-test. (TIF) [file pbio.1002177.s002.tif]

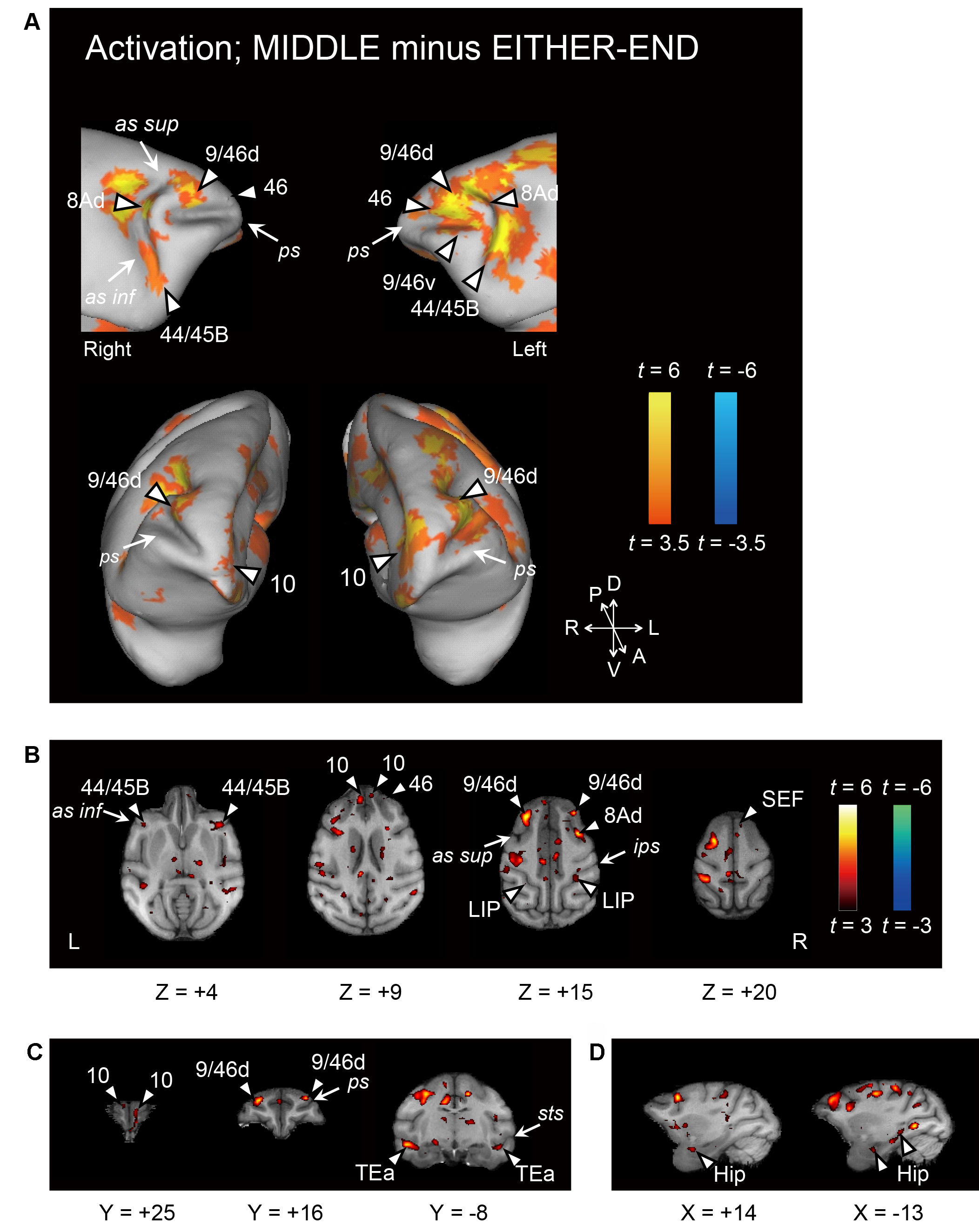

Supplement: S2 Fig — (A) Activity related to temporal-order judgment revealed by the contrast of MIDDLE minus EITHER-END. An activation map is superimposed on the inflated brain: top, lateral view; bottom, anterior view. (B–D) Activation map is superimposed on transverse sections (B), coronal sections (C), and sagittal sections (D). (TIF) [file pbio.1002177.s003.tif]

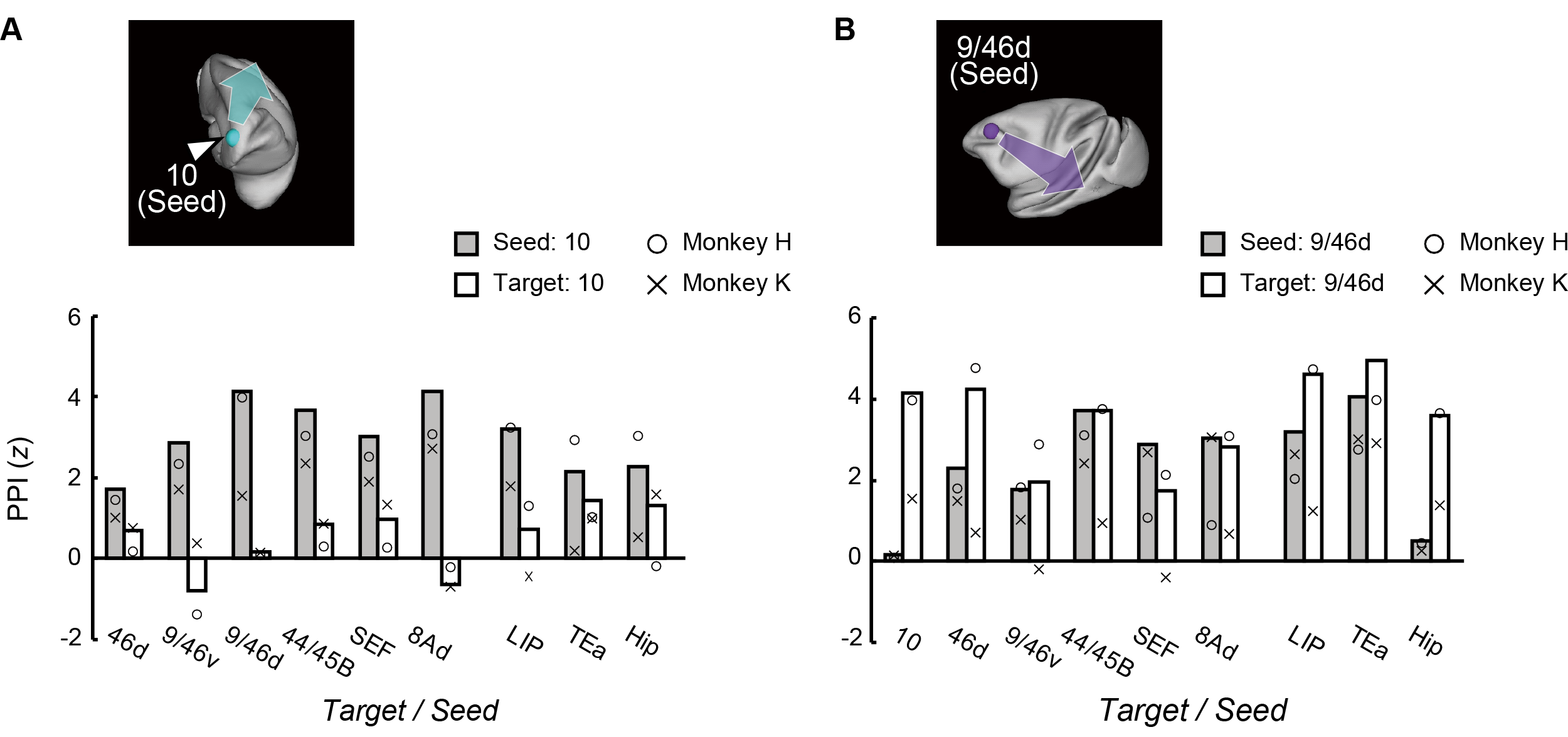

Supplement: S3 Fig — Corresponding data of individual monkeys for the data in Fig 3B and 3C. Each circle and cross represents data for monkey H and monkey K, respectively. Two bar plots in each column show z-values for PPIs from area 10 (A) or area 9/46d (B) to other ipsilateral homotopic areas (gray) and PPIs from other homotopic areas to area 10 (A) or area 9/46d (B) (white). (TIF) [file pbio.1002177.s004.tif]

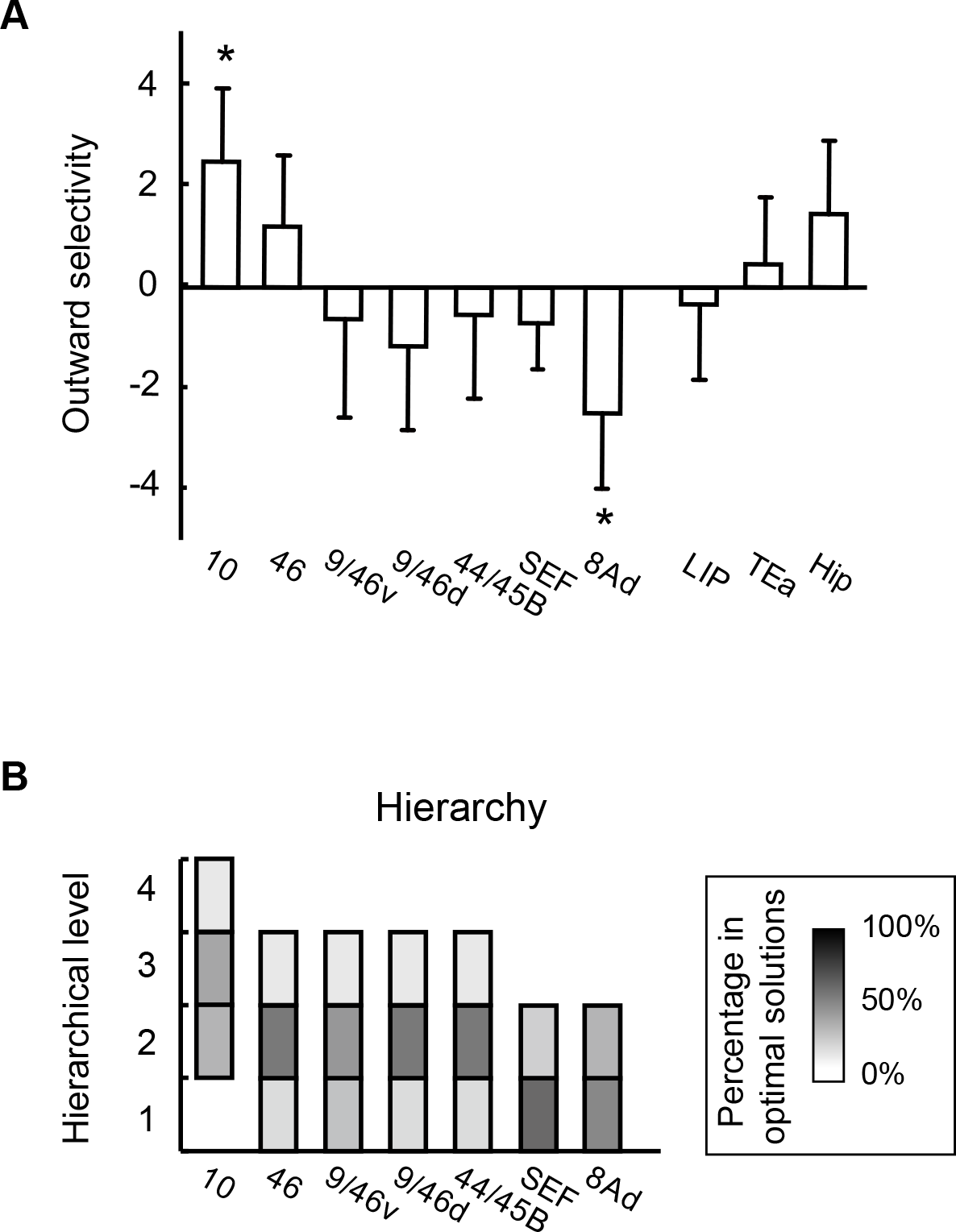

Supplement: S4 Fig — (A) Outward selectivities are plotted for each area. Error bars indicate SD. * p < 0.05 (Bonferroni correction). Area 10 has significant bias toward outward connectivity, whereas area 8Ad has significant bias toward inward connectivity. (B) Frequency distribution for 15 optimal hierarchical orderings in the PFC network. The boxes are shaded according to the relative occurrence of an area at a particular level across all the computed hierarchies. See “Hierarchical Structure in the Prefrontal Cortical Network” and “Estimation of Hierarchical Structure in the Task-Evoked Connectivity Network” in S1 Text. (TIF) [file pbio.1002177.s005.tif]

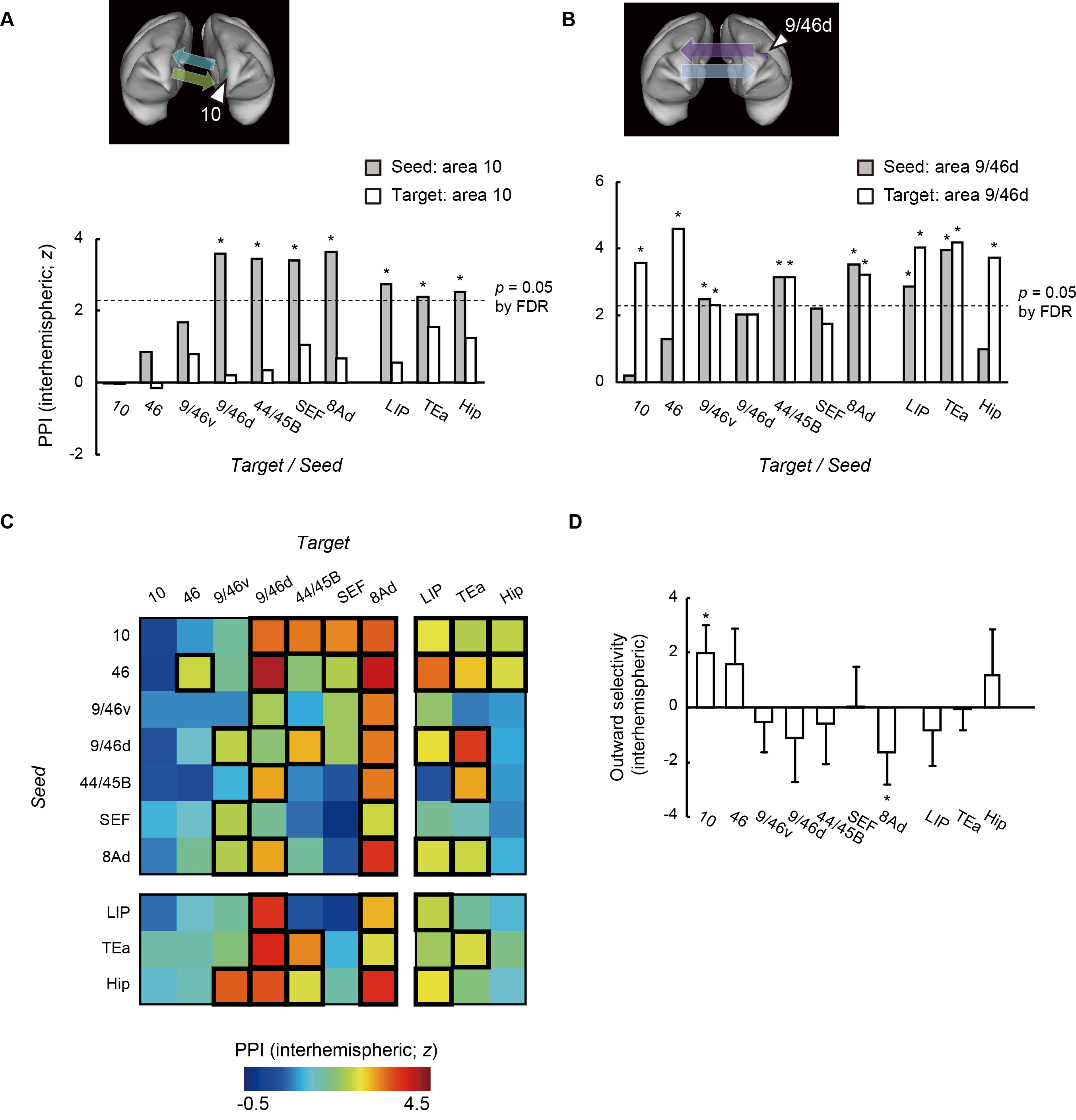

Supplement: S5 Fig — (A, B) Effects of interhemispheric PPI. Two bar plots in each column show z-values of interhemispheric PPIs from area 10 (A) or area 9/46d (B) to other homotopic areas (gray) and from other homotopic areas to area 10 (A) or area 9/46d (B) (white). Dashed lines indicate significance at p = 0.05 with FDR correction. * p < 0.05 with FDR correction. (C) Interhemispheric PPI matrix among the ten homotopic areas. Rows and columns indicate seed areas and target areas, respectively. Significant connectivities are enclosed by thick black lines (p < 0.05 with FDR correction). Intrahemispheric (see Fig 3D) and interhemispheric connectivity patterns were significantly correlated (r = 0.78, p = 1.1 × 10^−19^). (D) Outward selectivities are plotted for each area. Error bars indicate SD. * p < 0.05 with Bonferroni correction. (TIF) [file pbio.1002177.s006.tif]

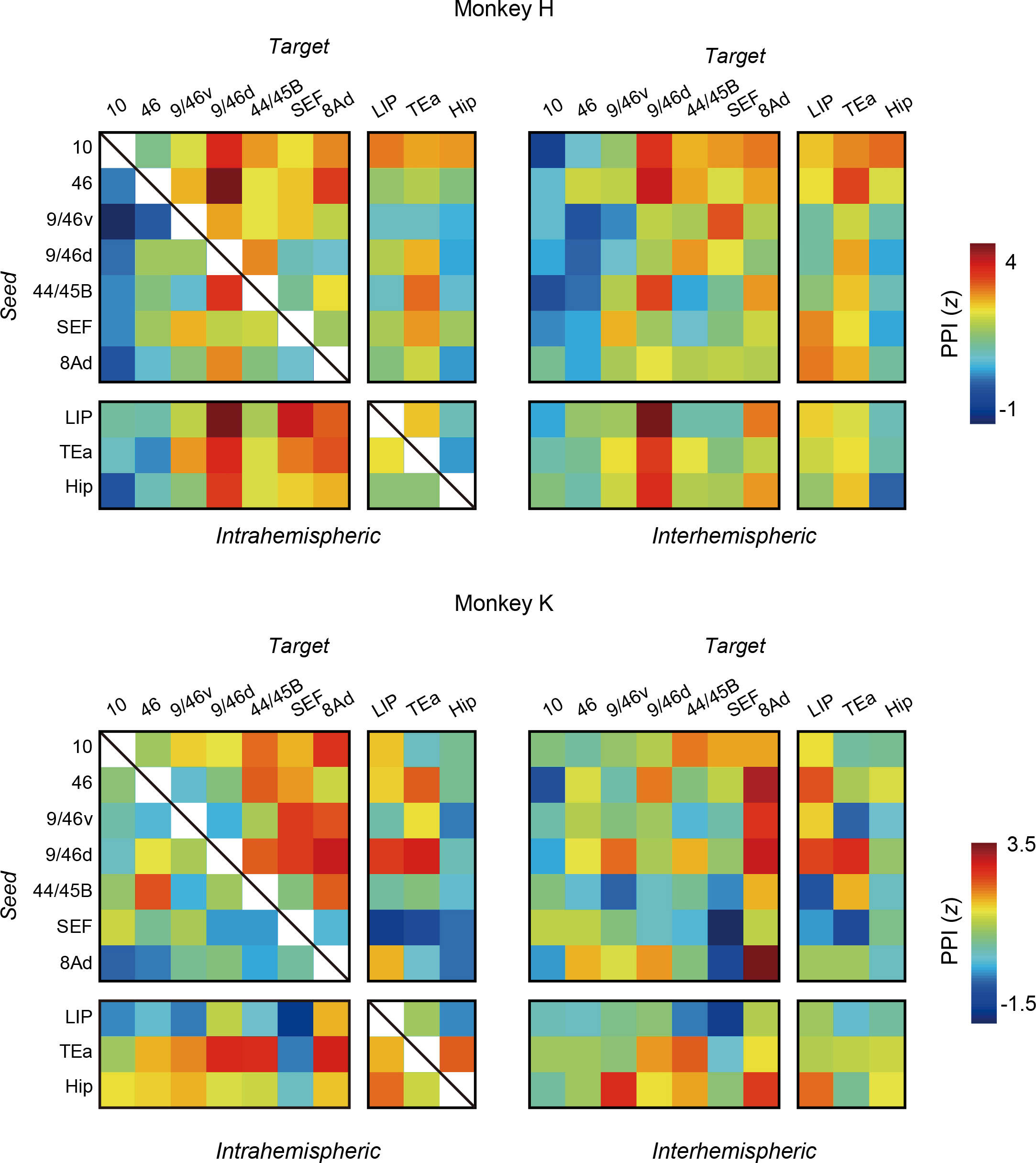

Supplement: S6 Fig — PPI matrix among the ten homotopic areas for each monkey (top, monkey H; bottom, monkey K; left, intrahemispheric connectivity; right, interhemispheric connectivity). Rows and columns indicate seed areas and target areas, respectively. A significant correlation was observed between two monkeys (r = 0.25, p = 0.004). (TIF) [file pbio.1002177.s007.tif]

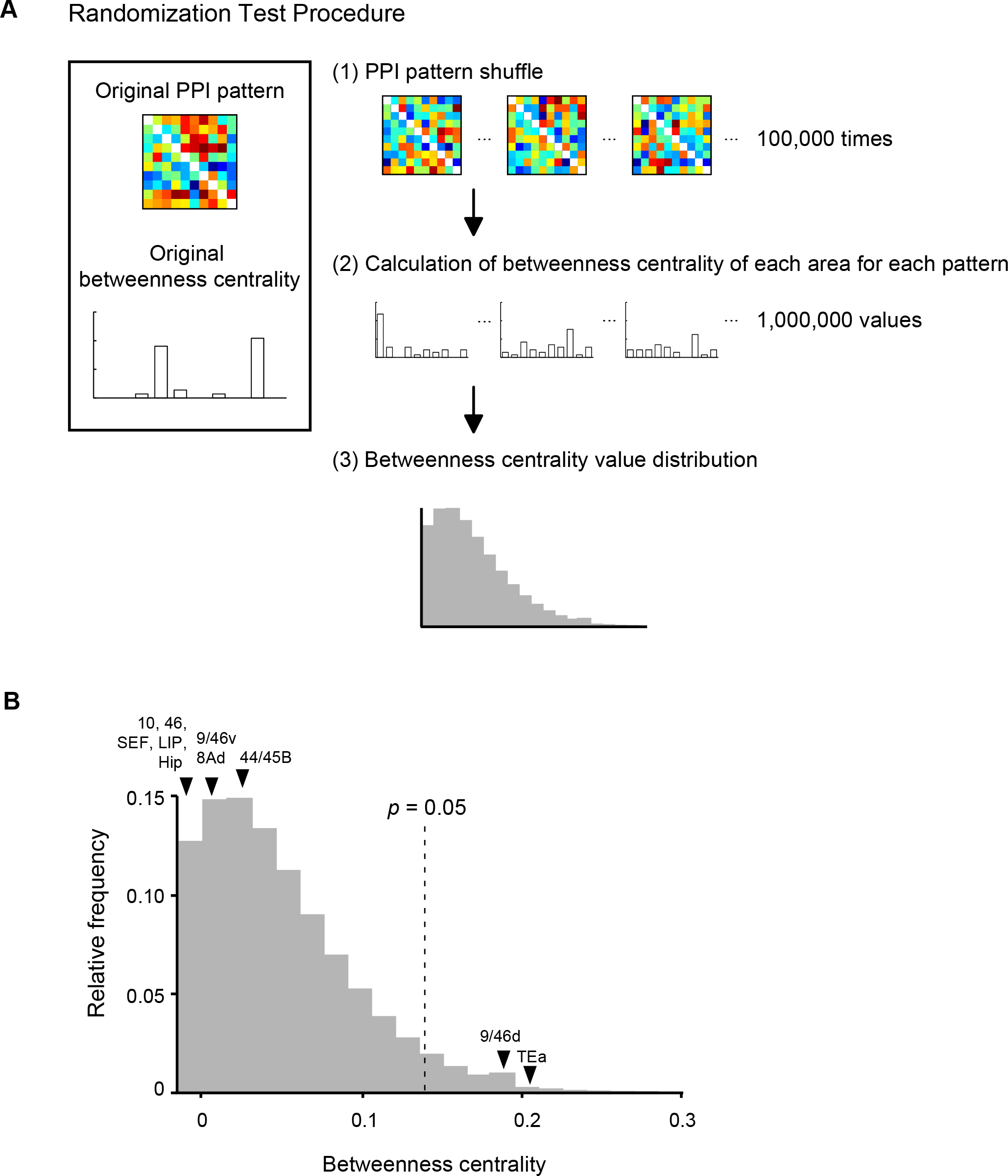

Supplement: S7 Fig — (A) Schematic illustration of randomization test procedure. (1) Randomly rewired connectivity patterns among the homotopic were generated 100,000 times by shuffling the original 90 connectivity values. (2) For each randomized network, we computed the betweenness centrality of each node. (3) The random distribution of betweenness centrality values were estimated by pooling all the values from all the nodes of all the randomized networks. (B) The positions of the actual measured betweenness centrality of each area in the computed distribution. Data of betweenness centrality from Fig 3E are shown. (TIF) [file pbio.1002177.s008.tif]

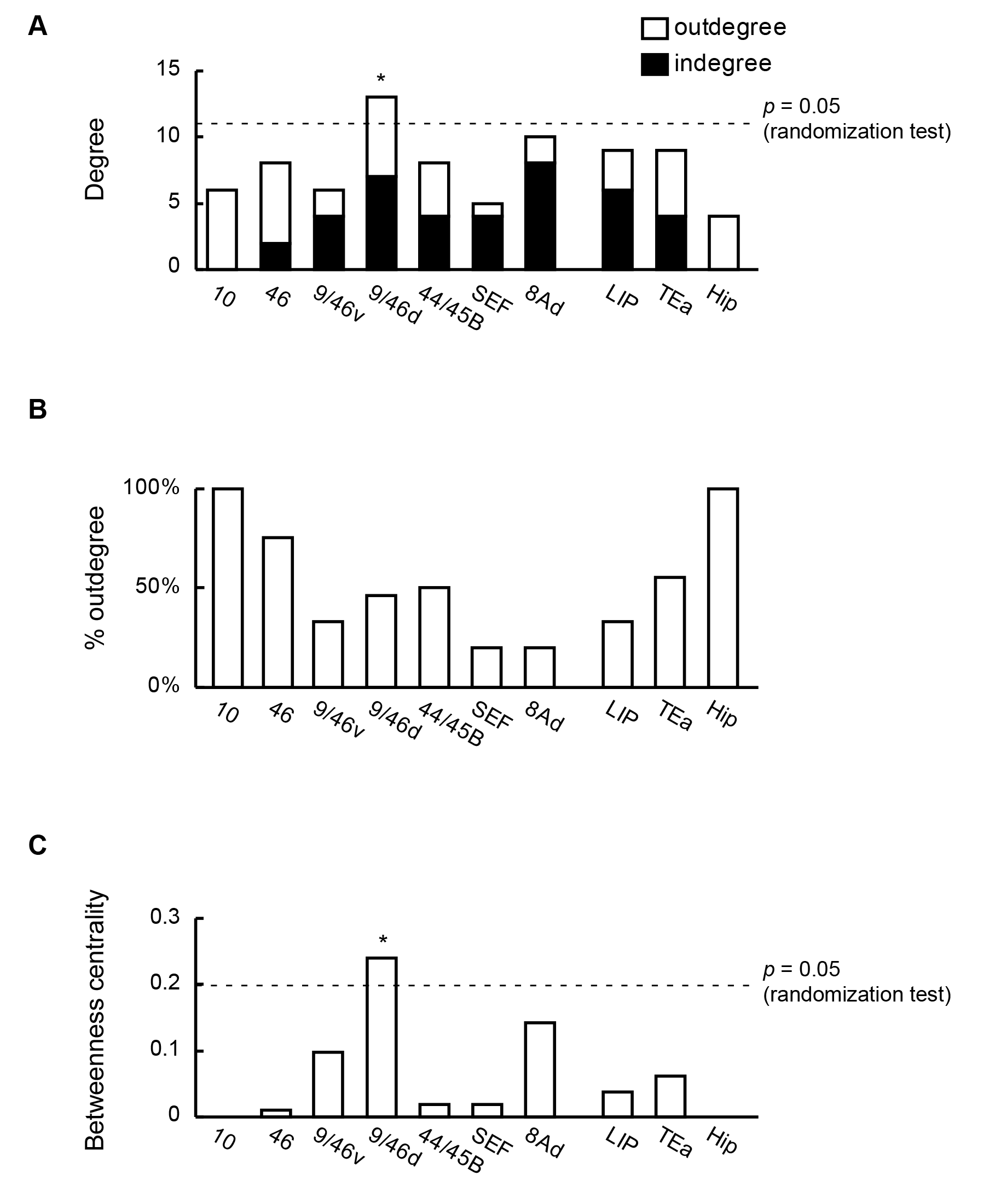

Supplement: S8 Fig — (A) Degrees (overall numbers of connections) are plotted for each area: white, outdegree (number of outward directed connections); black, indegree (number of inward directed connections). The dashed line indicates significance at p = 0.05 (randomization test). * p < 0.05. (B) Proportions of outdegree are plotted for each area. (C) Betweenness centralities are plotted for each area. The dashed line indicates significance at p = 0.05 (randomization test). * p < 0.05. (TIF) [file pbio.1002177.s009.tif]

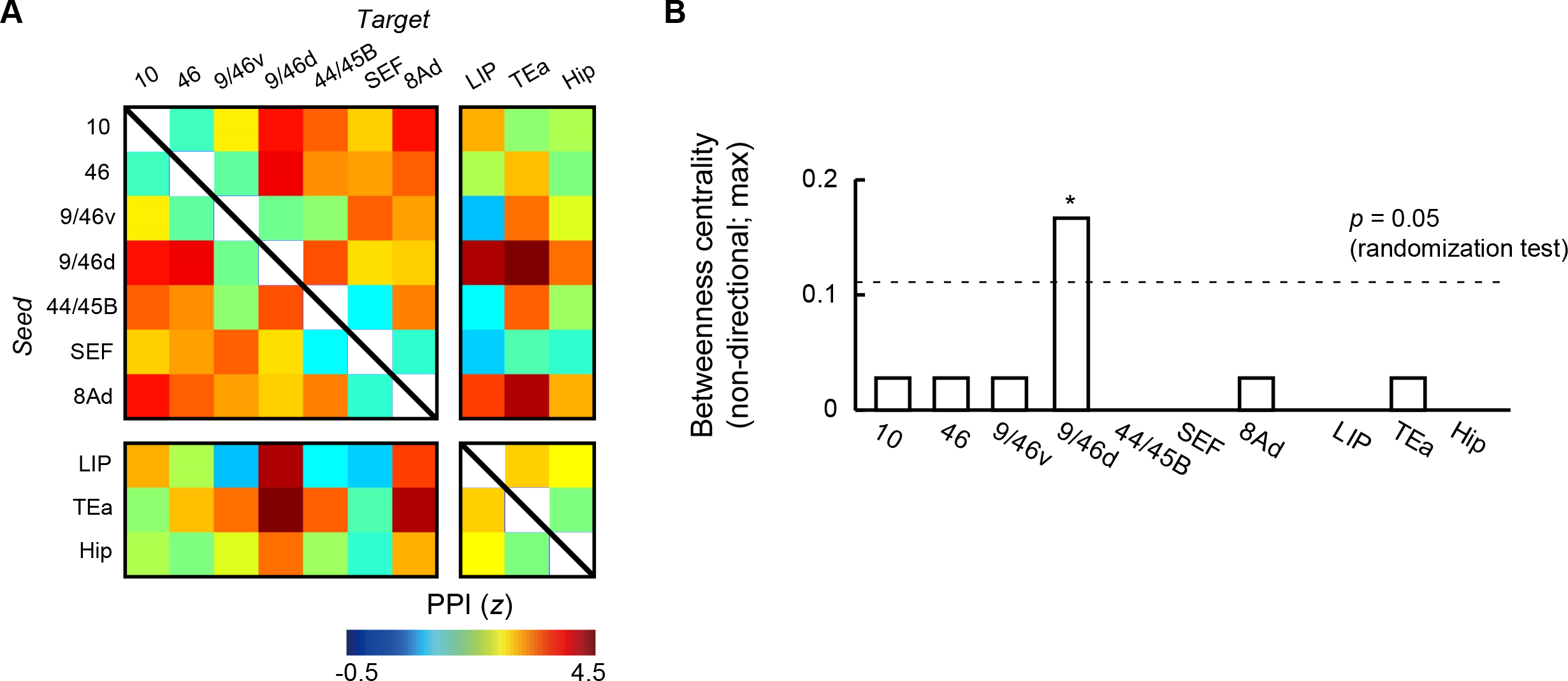

Supplement: S9 Fig — (A) PPI matrix among the homotopic areas without assumptions of directionality. The weight of the connection between A and B is evaluated as larger values of PPIA->B and PPIB->A. See also Fig 3D and 3F. (B) Betweenness centralities calculated based on (A). The dashed line indicates significance at p = 0.05 (randomization test). * p < 0.05. (TIF) [file pbio.1002177.s010.tif]

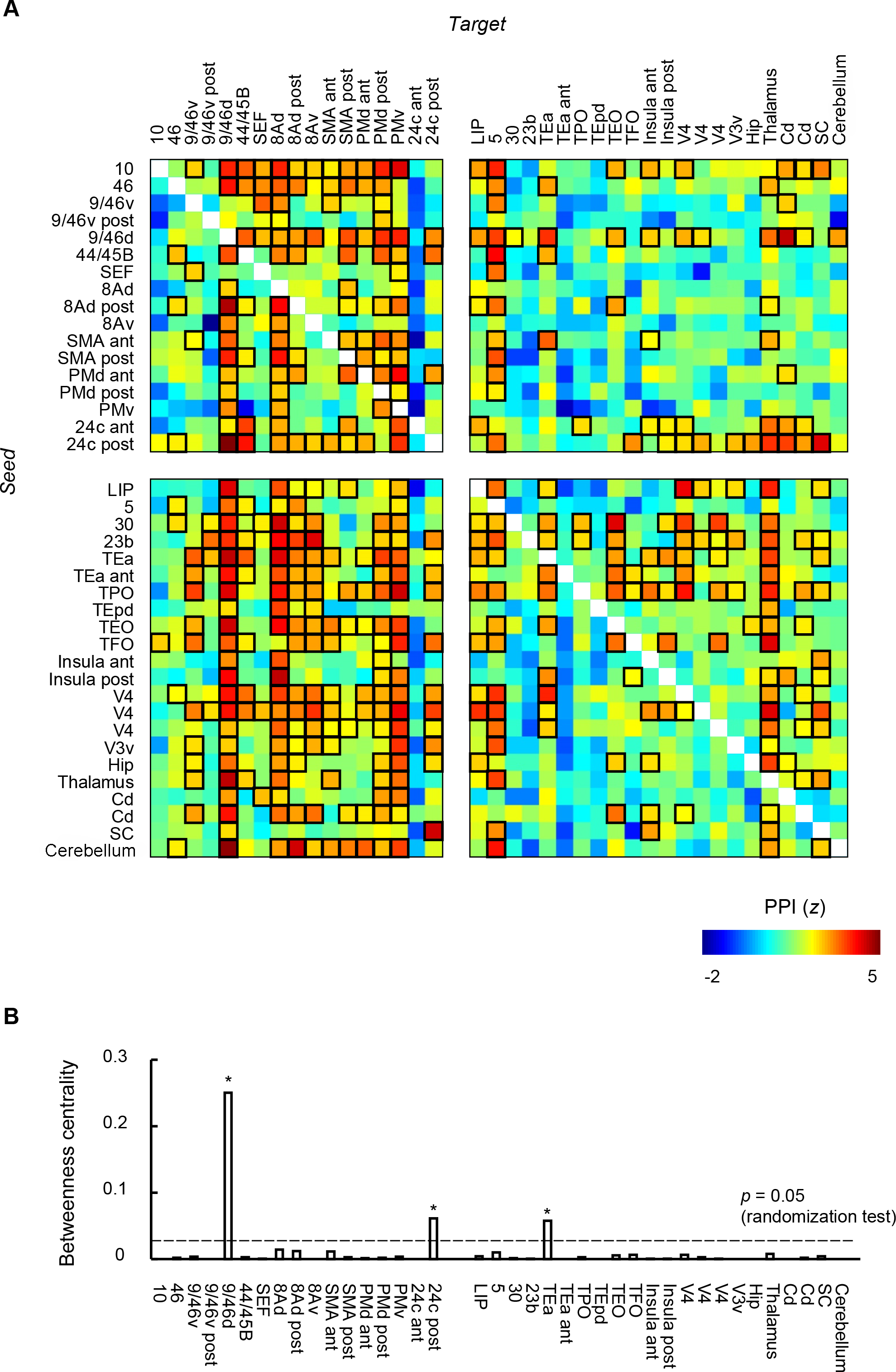

Supplement: S10 Fig — (A) PPI matrix among a set of 39 areas. Rows and columns indicate seed and target areas, respectively. Significant connectivities are enclosed by thick black lines (p < 0.05 with FDR correction). For both activation patterns and PPI connectivity patterns of the larger set, there was a significant correlation between two monkeys (for activation pattern, r = 0.26, p = 0.02; for PPI connectivity pattern, r = 0.15, p = 2.5 × 10^−17^). (B) Betweenness centralities are plotted for each area. The dashed line indicates significance at p = 0.05 (randomization test). * p < 0.05. (TIF) [file pbio.1002177.s011.tif]

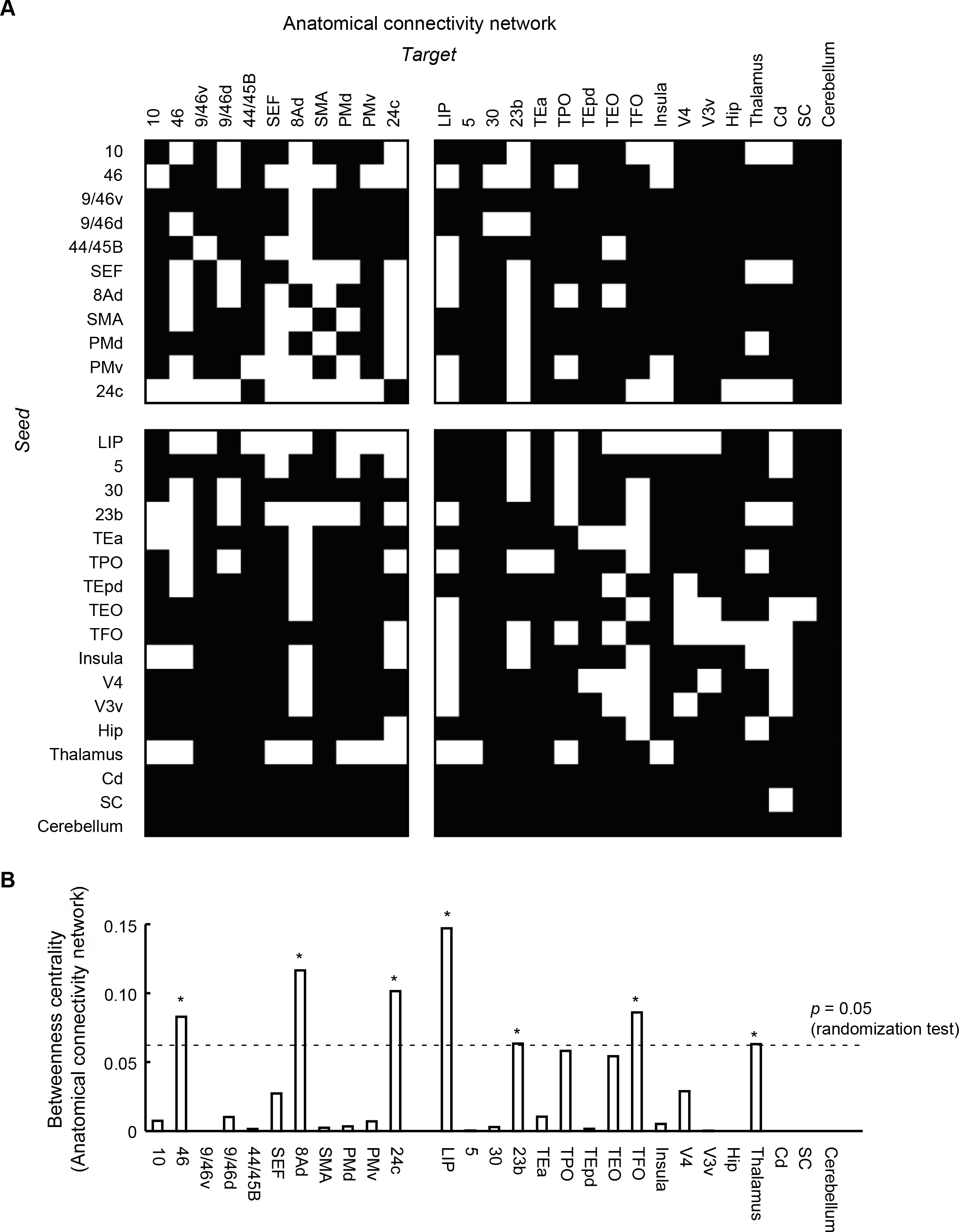

Supplement: S11 Fig — (A) Anatomical connectivity matrix among a larger set of areas. Rows and columns indicate seed and target areas, respectively. A white (black) square indicates the presence (absence) of anatomical connection from row to column. Anatomical information is based on the CoCoMac database [41,47,48]. The areas to which the same labels are given in CoCoMac database are merged respectively. The projections to/from areas 8Ad, SEF, PMd, PMv, 24c, LIP, 5, 23b, TEpd, TEO, and TFO listed in the matrix are categorized as those to/from areas 8A, 6DR, 6DC, 6VR, 24, POa, PE, 23, CITd, PIT, and TF in CoCoMac, respectively. (B) Betweenness centralities calculated based on (A). The dashed line indicates significance at p = 0.05 (randomization test). * p < 0.05. (TIF) [file pbio.1002177.s012.tif]

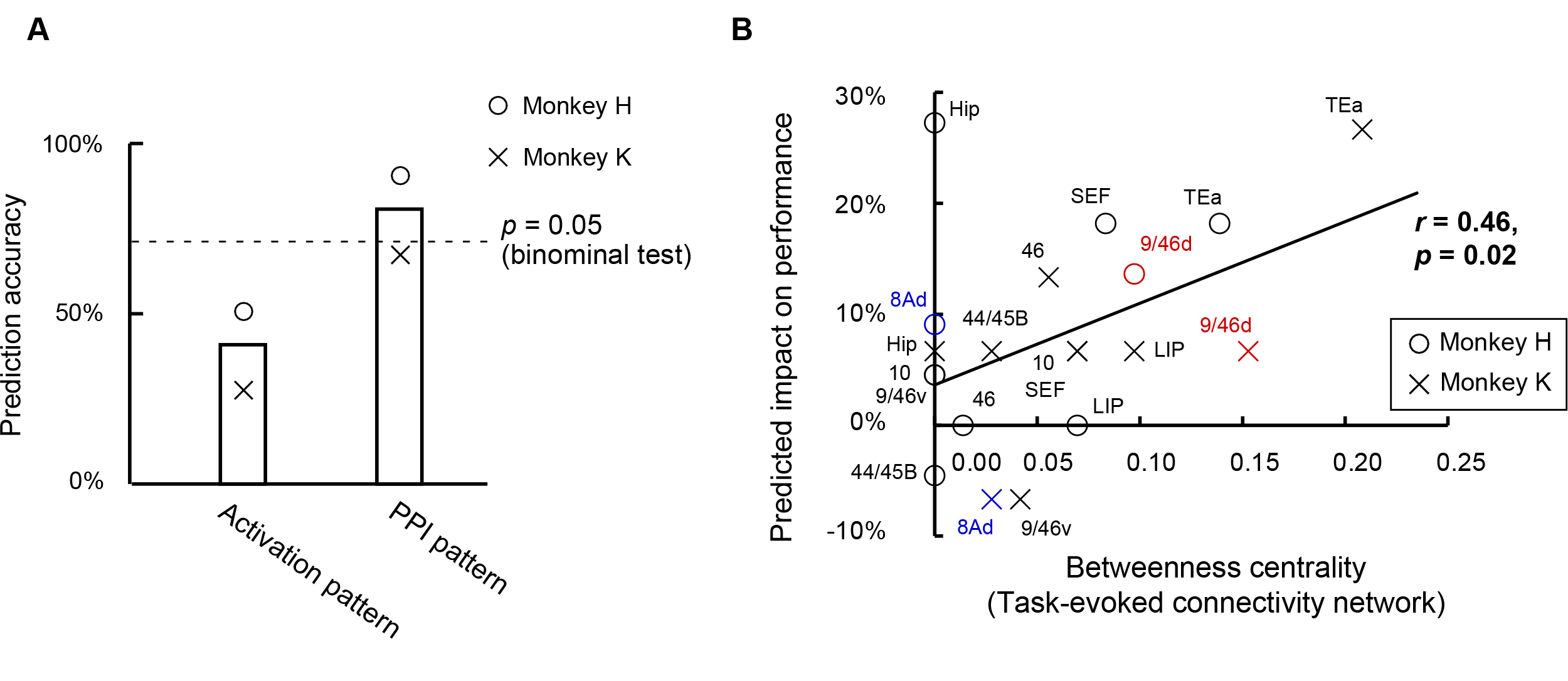

Supplement: S12 Fig — (A) Accuracy of behavioral performance prediction using activation patterns among the ten areas with ten features (left) and PPI connectivity patterns (right) with SVM analysis using linear kernels. The dashed line indicates accuracy significantly higher than chance (p = 0.05, binominal test, for group). Each circle and cross represents data for monkey H and monkey K, respectively. (B) Betweenness centrality calculated based on task-evoked connectivity (horizontal axis) and predicted impact on performance (vertical axis) estimated using linear kernels for each area for each monkey are plotted as a scattergram. The black line was fitted (r = 0.46, p = 0.02). (TIF) [file pbio.1002177.s013.tif]

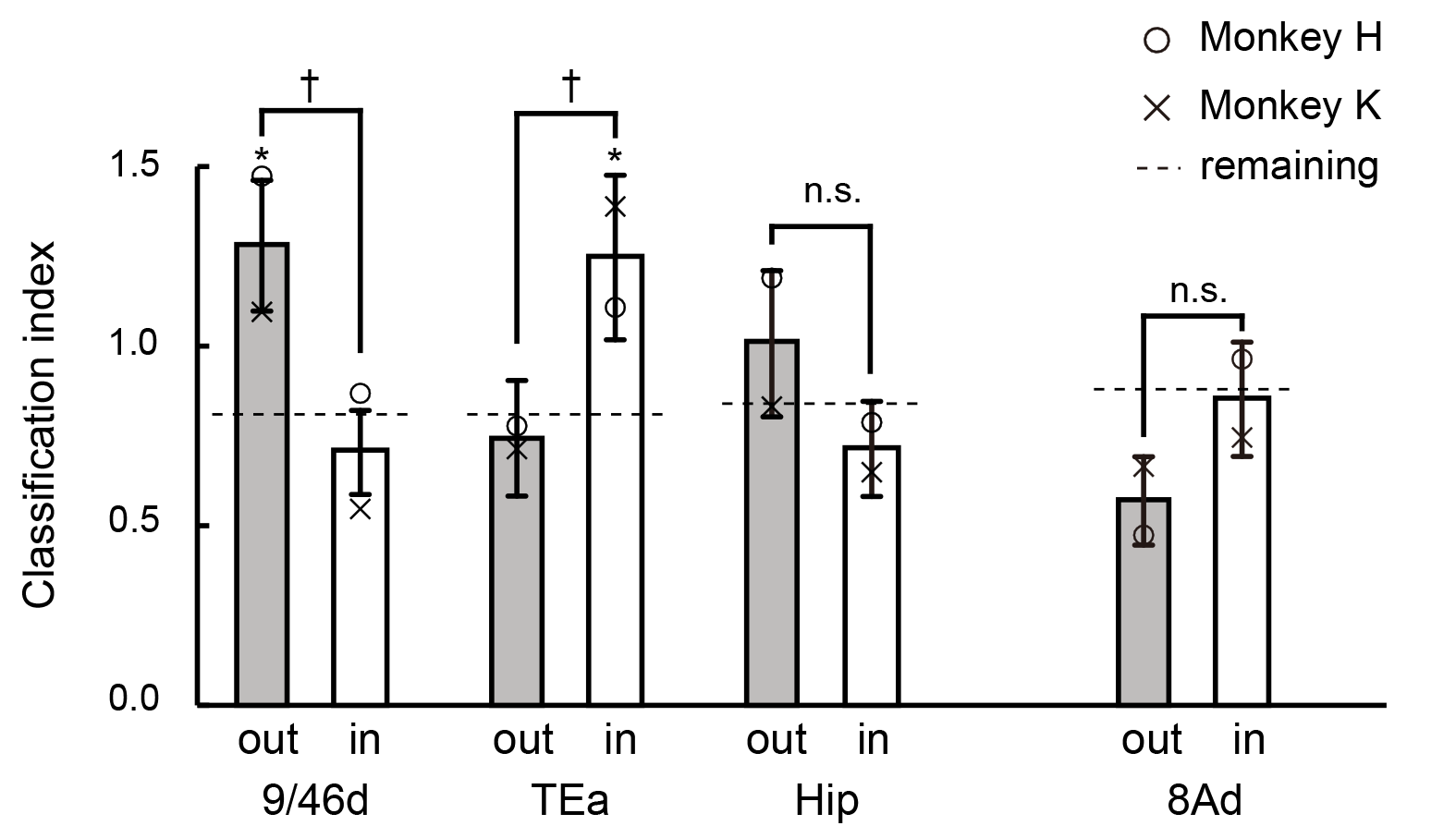

Supplement: S13 Fig — For areas 9/46d, TEa, and hippocampus, which showed significant predicted impact on performance, we compared the PPI values to and from deleted areas and estimated the contribution of inward and outward connectivity to the predicted impact on performance (“classification index”; see Materials and Methods). A three-way ANOVA (monkey × area [9/46d, TEa, hippocampus] × directionality [outward or inward]) of classification indices revealed no significant main effects of monkey, area, or directionality (all p > 0.2) or significant interactions between monkey and area or between monkey and directionality (all p > 0.3), though significant interaction between area and directionality was found (F(2, 96) = 5.00, p = 0.008). For area 9/46d, classification indices of outward directionality were significantly higher than those of inward directionality (p = 0.006, paired t-test) and were also significantly higher than the classification indices of other remaining connections (p = 0.008, t-test). For area TEa, classification indices of inward directionality were significantly higher than those of outward directionality (p = 0.04, paired t-test) and were also significantly higher than those of other remaining connections (p = 0.03, t-test). For hippocampus, there is a tendency for classification indices of outward directionality to be higher than those of inward directionality (p = 0.12, paired t-test). For comparison, in area 8Ad, there was no significant difference between the classification indices of outward and inward directionality and no significant increase of the classification indices of outward or inward directionality from those of other remaining connections (all p > 0.1). * p < 0.05, t-test. † p < 0.05, paired t-test. (TIF) [file pbio.1002177.s014.tif]

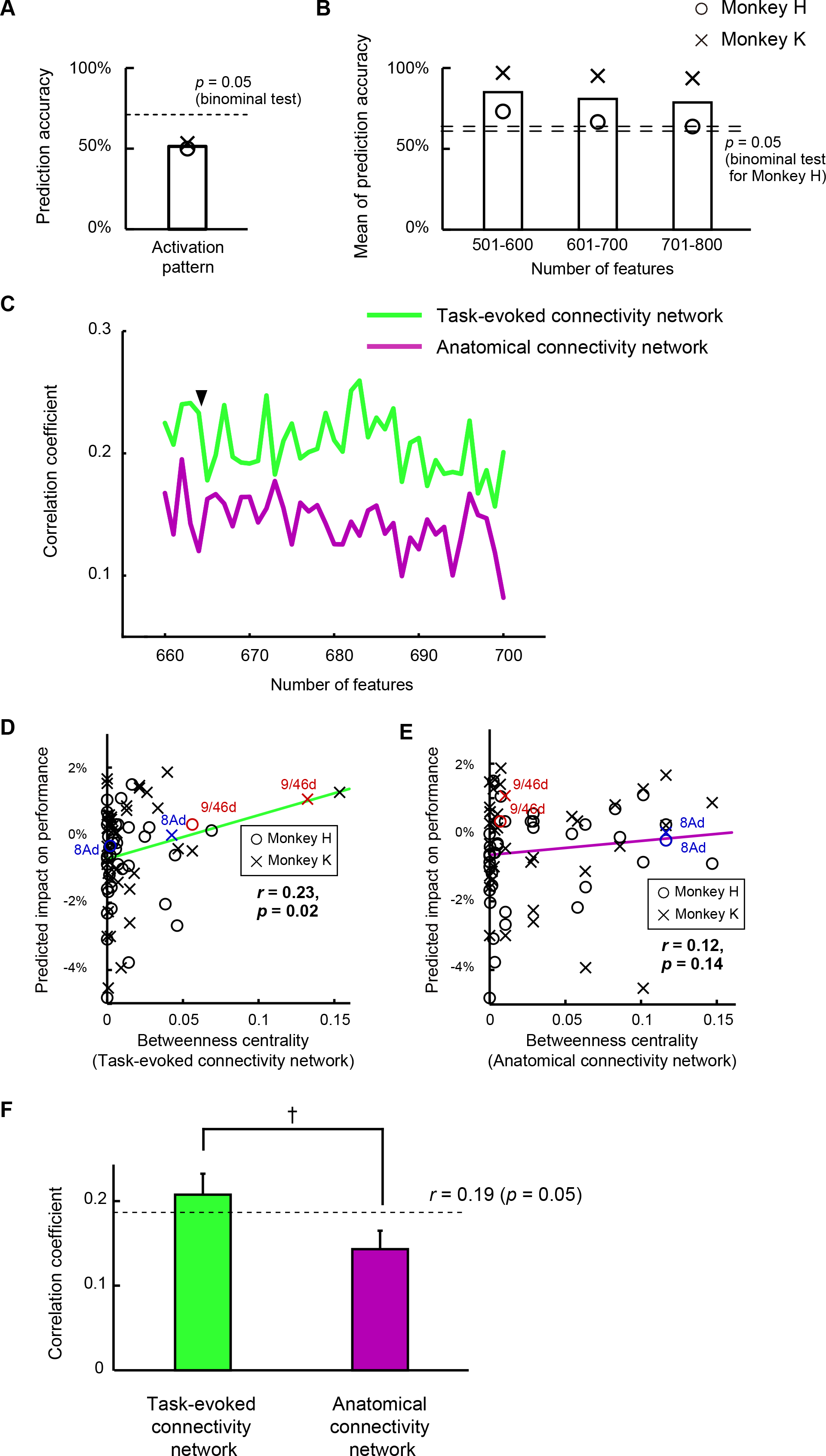

Supplement: S14 Fig — (A) Accuracy of behavioral performance prediction using activation patterns. The dashed line indicates accuracy significantly higher than chance (p = 0.05, binominal test, for group). The circle and the cross represents data for monkey H and monkey K, respectively. (B) Mean accuracy of behavioral performance prediction using PPI connectivity patterns after a “feature selection” procedure was plotted for the three ranges of number of selected features: 501 to 600 (left), 601 to 700 (middle), and 701 to 800 (right). The double dashed lines indicate accuracy for significantly higher than chance for monkey H (p = 0.05, binominal test). (C) Stability of correlation coefficient between predicted impact on performance and betweenness centrality as a function of the number of selected features. Betweenness centrality of each area was calculated based on task-evoked connectivity (green) and on anatomical connectivity (purple), respectively. (D) Betweenness centrality calculated based on task-evoked connectivity (horizontal axis) and predicted impact on performance (vertical axis) for each area for each monkey from the data point in (C) are plotted as a scattergram. The green line was fitted (r = 0.23, p = 0.02). (E) Betweenness centrality calculated based on anatomical connectivity (horizontal axis) and predicted impact on performance (vertical axis) for each area for each monkey from the data point in (C) are plotted as a scattergram. (F) Mean correlation coefficients between predicted impact on performance and betweenness centrality for the range of the number of selected features shown in (C). Betweenness centrality of each area was calculated based on task-evoked connectivity (green) and on anatomical connectivity (purple), respectively. A dashed line indicates a correlation coefficient of r = 0.19 (p = 0.05). † p < 10−16, paired t-test. Error bar indicates SD. (TIF) [file pbio.1002177.s015.tif]
