# Supplementary material for: Dynamically Allocated Hub in Task-Evoked Network Predicts the Vulnerable Prefrontal Locus for Contextual Memory Retrieval in Macaques
Source: PLoS Biol. 2015 Jun 30;13(6):e1002177. doi: 10.1371/journal.pbio.1002177 (PMC4488377; doi:10.1371/journal.pbio.1002177)
Supplement: S1 Table — Z-values of significant peaks for each monkey at a voxel level of p < 0.05 corrected by FWE within group homotopic regions. † Significant only at a voxel level of p < 0.05 corrected by FDR within group homotopic regions. ‡ Significant only at a voxel level of p < 0.05. (DOCX) [file pbio.1002177.s016.docx]

**S1 Table. Individual activation in homotopic areas.**

| Homotopic area | |  |  | *z* value for each subject | | |
| --- | --- | --- | --- | --- | --- | --- |
|  |  | Hemisphere |  | Monkey H |  | Monkey K |
| Frontal | |  |  |  |  |  |
|  | 10 | L | [ | 4.80 |  | 3.97 |
|  |  | R |  | 5.01 |  | 3.82 |
|  | 46 | L | [ | 4.47 |  | 3.22 |
|  |  | R |  | 5.28 |  | 2.75 |
|  | 9/46v | L | [ | 5.60 |  | 2.37† |
|  |  | R |  | 4.15 |  | 2.34† |
|  | 9/46d | L | [ | 4.43 |  | 3.28 |
|  |  | R |  | 4.74 |  | 3.53 |
|  | 44/45B | L | [ | 5.18 |  | 2.80 |
|  |  | R |  | 6.31 |  | 2.37† |
|  | SEF | L | [ | 3.67 |  | 4.31 |
|  |  | R |  | 4.55 |  | 3.46 |
|  | 8Ad | L | [ | 4.96 |  | 3.62 |
|  |  | R |  | 4.85 |  | 2.23† |
| Parietal | |  |  |  |  |  |
|  | LIP | L | [ | 4.49 |  | 3.70 |
|  |  | R |  | 5.43 |  | 1.75‡ |
| Temporal | |  |  |  |  |  |
|  | TEa | L | [ | 6.44 |  | 3.02 |
|  |  | R |  | 4.58 |  | 3.73 |
| Hippocampus | |  |  |  |  |  |
|  | Hip | L | [ | 4.96 |  | 2.43 |
|  |  | R |  | 4.19 |  | 3.41 |

*Z*-values of significant peaks for each monkey at a voxel level of *p* < 0.05 corrected by FWE within group homotopic regions. † Significant only at a voxel level of *p* < 0.05 corrected by FDR within group homotopic regions. ‡ Significant only at a voxel level of *p* < 0.05.
