# Supplementary material for: Dynamically Allocated Hub in Task-Evoked Network Predicts the Vulnerable Prefrontal Locus for Contextual Memory Retrieval in Macaques
Source: PLoS Biol. 2015 Jun 30;13(6):e1002177. doi: 10.1371/journal.pbio.1002177 (PMC4488377; doi:10.1371/journal.pbio.1002177)
Supplement: S2 Table — Significant peaks at a voxel level of p < 0.05 corrected by FDR. Coordinates are listed in monkey bicommissural space [26,28,34]. (DOCX) [file pbio.1002177.s017.docx]

**S2 Table. Reaction time-corrected activation in homotopic areas.**

| Homotopic area | |  |  | Coordinates (mm) | | |  |
| --- | --- | --- | --- | --- | --- | --- | --- |
|  |  | Hemisphere |  | X | Y | Z | *t* value |
| Frontal | |  |  |  |  |  |  |
|  | 10 | L | [ | -2 | 23 | 10 | 4.87 |
|  |  | R |  | 3 | 25 | 9 | 5.69 |
|  | 46 | L | [ | -11 | 18 | 12 | 4.91 |
|  |  | R |  | 9 | 22 | 11 | 5.73 |
|  | 9/46v | L | [ | -16 | 12 | 10 | 5.14 |
|  |  | R |  | 17 | 15 | 10 | 3.21 |
|  | 9/46d | L | [ | -12 | 11 | 16 | 4.64 |
|  |  | R |  | 12 | 16 | 15 | 3.30 |
|  | 44/45B | L | [ | -19 | 10 | 3 | 3.44 |
|  |  | R |  | 18 | 10 | 3 | 5.00 |
|  | SEF | L | [ | -7 | 11 | 21 | 3.28 |
|  |  | R |  | 3 | 9 | 20 | 2.78 |
|  | 8Ad | L | [ | -11 | 10 | 17 | 4.62 |
|  |  | R |  | 10 | 13 | 17 | 3.50 |
| Parietal | |  |  |  |  |  |  |
|  | LIP | L | [ | -12 | -19 | 16 | 3.82 |
|  |  | R |  | 14 | -16 | 15 | 3.39 |
| Temporal | |  |  |  |  |  |  |
|  | TEa | L | [ | -22 | -7 | -7 | 4.09 |
|  |  | R |  | 22 | -8 | -10 | 3.92 |
| Hippocampus | |  |  |  |  |  |  |
|  | Hip | L | [ | -12 | -3 | -10 | 2.78 |
|  |  | R |  | 13 | -2 | -11 | 2.70 |

Significant peaks at a voxel level of *p* < 0.05 corrected by FDR. Coordinates are listed in monkey bicommissural space [26, 28, 34].
