# Supplementary material for: Dynamically Allocated Hub in Task-Evoked Network Predicts the Vulnerable Prefrontal Locus for Contextual Memory Retrieval in Macaques
Source: PLoS Biol. 2015 Jun 30;13(6):e1002177. doi: 10.1371/journal.pbio.1002177 (PMC4488377; doi:10.1371/journal.pbio.1002177)
Supplement: S3 Table — Significant peaks at a voxel level of p < 0.05 corrected by FWE within group homotopic regions. Coordinates are listed in monkey bicommissural space [26,28,34]. (DOCX) [file pbio.1002177.s018.docx]

**S3 Table. Activation revealed by the contrast of MIDDLE minus EITHER-END in homotopic areas.**

| Homotopic area | |  |  | Coordinates (mm) | | |  |
| --- | --- | --- | --- | --- | --- | --- | --- |
|  |  | Hemisphere |  | X | Y | Z | *t* value |
| Frontal | |  |  |  |  |  |  |
|  | 10 | L | [ | -2 | 22 | 11 | 4.64 |
|  |  | R |  | 2 | 25 | 8 | 3.77 |
|  | 46 | L | [ | -11 | 17 | 13 | 4.10 |
|  |  | R |  | 9 | 22 | 10 | 3.54 |
|  | 9/46v | L | [ | -13 | 15 | 13 | 4.69 |
|  |  | R |  | 16 | 15 | 10 | 2.58 |
|  | 9/46d | L | [ | -12 | 15 | 14 | 5.68 |
|  |  | R |  | 12 | 16 | 15 | 5.11 |
|  | 44/45B | L | [ | -19 | 10 | 4 | 3.66 |
|  |  | R |  | 18 | 10 | 3 | 4.48 |
|  | SEF | L | [ | -6 | 12 | 21 | 3.56 |
|  |  | R |  | 4 | 10 | 20 | 3.23 |
|  | 8Ad | L | [ | -11 | 10 | 16 | 5.02 |
|  |  | R |  | 9 | 12 | 15 | 3.12 |
| Parietal | |  |  |  |  |  |  |
|  | LIP | L | [ | -12 | -19 | 16 | 3.99 |
|  |  | R |  | 13 | -17 | 16 | 4.01 |
| Temporal | |  |  |  |  |  |  |
|  | TEa | L | [ | -24 | -8 | -8 | 5.13 |
|  |  | R |  | 22 | -8 | -9 | 3.96 |
| Hippocampus | |  |  |  |  |  |  |
|  | Hip | L | [ | -12 | -3 | -10 | 3.03 |
|  |  | R |  | 13 | -2 | -11 | 4.13 |

Significant peaks at a voxel level of *p* < 0.05 corrected by FWE within group homotopic regions. Coordinates are listed in monkey bicommissural space [26, 28, 34].
