# Supplementary material for: Dynamically Allocated Hub in Task-Evoked Network Predicts the Vulnerable Prefrontal Locus for Contextual Memory Retrieval in Macaques
Source: PLoS Biol. 2015 Jun 30;13(6):e1002177. doi: 10.1371/journal.pbio.1002177 (PMC4488377; doi:10.1371/journal.pbio.1002177)
Supplement: S4 Table — Significant peaks at a voxel level of p < 0.05 corrected by FWE. Coordinates are listed in monkey bicommissural space [26,28,34]. † Significant only at a voxel level of p < 0.001. (DOCX) [file pbio.1002177.s019.docx]

**S4 Table. Activations in homotopic areas in delayed matching-to-sample task.**

| Homotopic area | |  |  | Coordinates (mm) | | |  |
| --- | --- | --- | --- | --- | --- | --- | --- |
|  |  | Hemisphere |  | X | Y | Z | *t* value |
| Frontal | |  |  |  |  |  |  |
|  | 9/46v | L | [ | -19 | 15 | 8 | 5.51 |
|  |  | R |  | 19 | 13 | 10 | 3.88† |
|  | 8Ad | L | [ | -15 | 7 | 12 | 5.75 |
|  |  | R |  | 12 | 5 | 11 | 4.25† |
|  | 11 | L | [ | -11 | 16 | 7 | 5.13 |
|  |  | R |  | 12 | 16 | 7 | 4.37† |
|  | PMd | L | [ | -10 | 10 | 18 | 5.08 |
|  |  | R |  | 8 | 8 | 16 | 5.85 |
| Parietal | |  |  |  |  |  |  |
|  | PG | L | [ | -21 | -25 | 13 | 6.42 |
|  |  | R |  | 21 | -23 | 13 | 5.69 |
|  | LIP | L | [ | -14 | -18 | 20 | 5.84 |
|  |  | R |  | 12 | -19 | 17 | 4.87 |
| Temporal | |  |  |  |  |  |  |
|  | AITv | L | [ | -17 | -1 | -16 | 5.06 |
|  |  | R |  | 18 | -1 | -16 | 3.91† |
|  | AITd ant | L | [ | -21 | -4 | -14 | 5.04 |
|  |  | R |  | 23 | -3 | -14 | 6.37 |
|  | AITd post | L | [ | -26 | -7 | -12 | 6.56 |
|  |  | R |  | 26 | -7 | -15 | 4.92 |
|  | TEm | L | [ | -27 | -13 | -3 | 8.37 |
|  |  | R |  | 27 | -12 | -5 | 4.69† |
|  | TEO ant | L | [ | -24 | -19 | 6 | 7.17 |
|  |  | R |  | 26 | -17 | 4 | 5.35 |
|  | TEO post | L | [ | -27 | -20 | -2 | 7.64 |
|  |  | R |  | 28 | -19 | -4 | 6.72 |
| Occipital | |  |  |  |  |  |  |
|  | V2 | L | [ | -24 | -27 | 7 | 9.33 |
|  |  | R |  | 23 | -29 | 5 | 6.73 |
|  | V2 | L | [ | -26 | -26 | 0 | 8.8 |
|  |  | R |  | 25 | -25 | 2 | 6.98 |
|  | V2 | L | [ | -22 | -31 | -1 | 7.22 |
|  |  | R |  | 24 | -30 | 0 | 6.79 |

Significant peaks at a voxel level of *p* < 0.05 corrected by FWE. Coordinates are listed in monkey bicommissural space [26, 28, 34]. † Significant only at a voxel level of *p* < 0.001.
