# Supplementary material for: Dynamically Allocated Hub in Task-Evoked Network Predicts the Vulnerable Prefrontal Locus for Contextual Memory Retrieval in Macaques
Source: PLoS Biol. 2015 Jun 30;13(6):e1002177. doi: 10.1371/journal.pbio.1002177 (PMC4488377; doi:10.1371/journal.pbio.1002177)
Supplement: S1 Text — (DOCX) [file pbio.1002177.s020.docx]

**Supporting Information**

**S1 Text**

**Supplementary Results**

**Behavioral Effects due to the Inclusion of End Stimuli**

In order to evaluate the stability of the strategy to solve the temporal-order judgment task, two other stimuli in the same list were presented as a follow-up choice pair after correct responses in certain trials (see Materials and Methods). The differences in correct response rates and reaction times due to the inclusion of end stimuli (as shown in Fig 1B) were reproduced in these follow-up choice pairs (for correct response rate, 63.7 ± 10.4 %, 83.6 ± 6.2 %, and 95.8 ± 4.1 % [mean ± SD] in MIDDLE, EITHER-END and BOTH-END trials, respectively; for reaction time, 1192 ± 250 ms, 970 ± 191 ms, and 861 ± 206 ms [mean ± SD] in MIDDLE, EITHER-END and BOTH-END trials, respectively; all [MIDDLE vs. EITHER-END, MIDDLE vs. BOTH-END] *p* < 10^-8^, paired *t*-test). These results confirmed the differences in cognitive demands for temporal-order judgment that vary depending on whether the end stimuli are included or not.

**Hierarchical Structure in the Prefrontal Cortical Network**

In the task-evoked connectivity network among the activated PFC areas (Fig 3D), significant PPIs from area 10 were observed in a majority of other PFC areas (Fig 3B, gray; for individual monkey data, S3A Fig), whereas no significant PPIs from other areas were found in area 10 (Fig 3B, white). On the other hand, significant PPIs were found in connectivities both to and from area 9/46d (Fig 3C; for individual monkey data, S3B Fig). We estimated the hierarchical structure (see “Estimation of Hierarchical Structure in the Task-evoked Connectivity Network” in S1 Text). The results showed that area 10 had significant bias toward outward connectivity (“outward selectivity”, *t*(8) = 5.19, *p* = 0.008 [Bonferroni correction], paired *t*-test) (S4A Fig) and was situated at the highest level in the hierarchical structure among the PFC areas (S4B Fig). These results were consistent with the proposed function of the human frontal pole [1, 2], medial part of which functionally corresponds to macaque area 10 [3].

**Prediction with the Network with a Larger Set of Areas**

We expanded the SVM analysis to a larger set of areas (total 39 areas) (see Table 1 and S10 Fig). First, SVM classification analysis using activation patterns was conducted. Even with the larger set, activation patterns across the areas failed to predict behavioral performance of monkeys (all *p* > 0.1, binominal test) (S14A Fig), which is consistent with the results that were demonstrated using activation patterns across the ten homotopic areas (see Fig 5B). Next we conducted the SVM analysis using the PPI connectivity pattern among the larger set of areas. Because the number of connectivities among the 39 areas (i.e., 39 × 38 = 1482) is much larger than the number of sessions, we conducted a “feature selection” procedure according to the standard method before SVM analysis (see “SVM Analysis Using a Larger Set of Areas” in S1 Text) [4-6]. After selection of features up to 700, PPI patterns across areas could accurately predict session-by-session performance in both monkeys (*p* < 0.05, binominal test) (feature number range: 501-600, 601-700) (S14B Fig). Therefore, we focused on the selected feature number within the range of 660 to 700 (see “SVM Analysis Using a Larger Set of Areas” in S1 Text), and then calculated correlation coefficient between predicted impact on performance and betweenness centrality. Betweenness centrality was calculated based on task-evoked connectivity and on anatomical connectivity, respectively (S14C, D, E, F Fig). The predicted impact on performance correlated more highly with betweenness centrality based on task-evoked connectivity than with betweenness centrality based on anatomical connectivity, regardless of the number of selected features (*p* = 8.8 × 10^-17^, paired *t*-test) (S14F Fig). These observations are consistent with the results that were demonstrated using SVM analysis with the ten homotopic areas (see Fig 6A, 6B).

**Supplementary Methods**

**Estimation of Hierarchical Structure in the Task-Evoked Connectivity Network**

To quantitatively estimate the bias toward outward connectivity, we compared the outward PPIs to other areas with inward PPIs from other areas for each area and defined the mean difference between *z*-values as “outward selectivity”. A larger value indicates that an area tended to have a higher bias toward outward connectivity.

We estimated the hierarchical levels of seven areas in the prefrontal network. Based on the connection patterns in the binarized network (thresholded at *p* < 0.05 with FDR correction), hierarchical ordering was estimated based on the computational approach adopted in the previous study [7] using the following constraint: if a significant PPI connectivity from area *A* to area *B* exists, the level of *A* is higher than or equal to that of *B* (Level[*A*] ≥ Level[*B*]). Optimal solutions that fit the data permitting zero or one violation were identified among the possible orderings. A trivial solution that all the levels were the same was excluded from the optimal solutions. Among the different optimal hierarchical orderings of the areas, frequency distribution was calculated (see S2 Text for the code).

**SVM Analysis Using a Larger Set of Areas**

We conducted the SVM analysis using the PPI connectivity pattern among the larger set of areas including non-homotopic areas (total 39 areas; see Table 1). Because the number of connectivities (or, features) among the 39 areas (or, nodes) (i.e., 39 × 38 = 1482) is much larger than the number of sessions (or, samples), we conducted “feature selection” procedure according to the standard method before SVM analysis [4-6]. We applied a *t*-value filter for feature selection based on each feature’s *t*-value for the difference in PPI values between high and low performance sets. After *t*-value filter, the top *N* features were selected for each number of selected features *N* based on the ranking of *t*-values (see S2 Text for the code). Then, to examine the effect of node deletion on prediction accuracy of behavioral performance, we determined the number of features to use for the SVM analysis by the following criteria: 1) minimum number of connections to/from one node was more than 30% of the total number of connections, and 2) mean prediction accuracy was more than 80%. We calculated the predicted impact on performance for each node, and correlation coefficient between the predicted impact on performance and betweenness centrality (see S2 Text for the code).

**Data Analysis of Eye Movement**

We analyzed the eye movement data during temporal-order judgment from one monkey. The eye movement data from 500 ms before response onset to 500 ms after response onset were analyzed. We compared average eye positions during the period between MIDDLE conditions and BOTH-END conditions for both laterality (trials when the left choice was correct, and the other trials when the right choice was correct) (*t*-test). We also compared fluctuation of eye positions (SD of the eye positions during the period) between MIDDLE and BOTH-END conditions using two-way analysis of variance (ANOVA) (trial condition [MIDDLE vs BOTH-END] × laterality [left correct vs right correct]).

**References**

1. Passingham RE, Wise SP. (2012) The neurobiology of the prefrontal cortex: Anatomy, evolution, and the origin of insight. Oxford: Oxford University Press.
2. Badre D, D'Esposito M. (2009) Is the rostro-caudal axis of the frontal lobe hierarchical? Nat Rev Neurosci 10(9): 659-669.
3. Neubert FX, Mars RB, Thomas AG, Sallet J, Rushworth MF. (2014) Comparison of human ventral frontal cortex areas for cognitive control and language with areas in monkey frontal cortex. Neuron 81(3): 700-713.
4. Guyon I, Elisseeff A. (2003) An introduction to variable and feature selection. The Journal of Machine Learning Research 3: 1157-1182.
5. De Martino F, Valente G, Staeren N, Ashburner J, Goebel R, et al. (2008) Combining multivariate voxel selection and support vector machines for mapping and classification of fMRI spatial patterns. Neuroimage 43(1): 44-58.
6. Dosenbach NU, Nardos B, Cohen AL, Fair DA, Power JD, et al. (2010) Prediction of individual brain maturity using fMRI. Science 329(5997): 1358-1361.
7. Hilgetag CC, O'Neill MA, Young MP. (1996) Indeterminate organization of the visual system. Science 271(5250): 776-777.
