# Supplementary material for: Dynamically Allocated Hub in Task-Evoked Network Predicts the Vulnerable Prefrontal Locus for Contextual Memory Retrieval in Macaques
Source: PLoS Biol. 2015 Jun 30;13(6):e1002177. doi: 10.1371/journal.pbio.1002177 (PMC4488377; doi:10.1371/journal.pbio.1002177)
Supplement: S2 Text — (DOCX) [file pbio.1002177.s021.docx]

**Supporting Information**

**S2 Text**

**Matlab-style codes**

1. Network analyses:
2. Calculations of betweenness centrality in PPI *z*-value matrix, binarized matrix from PPI *z*-value matrix, and degrees and betweenness centrality in the binarized matrix (Fig 3D, 3E, S8 Fig)
3. Calculations of non-directional matrix from PPI *z*-value matrix and betweenness centrality in non-directional matrix (Fig 3F, 3G, S9 Fig)
4. Estimation of hierarchical levels of network nodes (S4B Fig)
5. SVM analyses:
6. Performance prediction analysis (Fig 5B)
7. Analysis of predicted impact on performance (Fig 5C)
8. Performance prediction analysis for a larger set of nodes (S14B Fig)
9. Analysis of predicted impact on performance for a larger set of nodes (S14D, E Fig)

(A-1)

%%%%%%%%%%%%%%%%%%%%%%%%%%%%%%%%%%%%%%%%%%%%%

% Matlab-style code for

% - calculation of binarized matrix from PPI z-value matrix

% - calculation of degrees from binarized matrix

% - calculation of betweenness centrality from these matrices

% (Fig 3D, 3E, S8 Fig)

function [wBin, inDegree, outDegree, bcDistZ, bcBin]...

= betweennessCentralityPPI(zValue, threshold)

% <betweennessCentralityPPI> requires N x N matrix <zValue> containing

% PPI values, and <threshold> for binarization of <zValue>.

% <wBin> is generated by binarizing <zValue>.

% <inDegree> and <outDegree> are indegree and outdegree of each node

% in <wBin>, respectively.

% <bcDistZ> is betweenness centrality of distance matrix

% defined from <zValue>, and <bcBin> is betweenness centrality

% of <wBin>.

%

% Requirement: Brain Connectivity Toolbox

% https://sites.google.com/site/bctnet/

wBin = (zValue is binarized depending on whether each PPI value is over <threshold> or not);

% Distance is defined as the inverse of z value

distZ = (each component of zValue is inversed);

% Degree of binarized matrix

% Brain Connectivity Toolbox (https://sites.google.com/site/bctnet/) is required

[inDegree, outDegree, degree] = degrees_dir(wBin);

% Betweenness centrality

% Brain Connectivity Toolbox (https://sites.google.com/site/bctnet/) is required

bcDistZ = betweenness_wei(distZ)/((N-1)*(N-2));

bcBin = betweenness_bin(wBin)/((N-1)*(N-2));

%%%%%%%%%%%%%%%%%%%%%%%%%%%%%%%%%%%%%%%%%%%%%

(A-2)

%%%%%%%%%%%%%%%%%%%%%%%%%%%%%%%%%%%%%%%%%%%%%

% Matlab-style code for

% - calculation of non-directional matrix from PPI z-value matrix

% - calculation of betweenness centrality from non-directional matrix

% (Fig 3F, 3G, S9 Fig)

function [zMean, zMax, bcDistZMean, bcDistZMax]...

= betweennessCentralityNondirectionalMatrix(zValue)

% <betweennessCentralityNondirectionalMatrix> requires N x N matrix

% <zValue> containing PPI values.

% <zMean> (and <zMax>) is a non-directional matrix generated by taking

% mean (and max) of <zValue> and its transpose.

% <bcDistZMean> and <bcDistZMax> are betweenness centralities

% of distance matrices defined from <zMean>, and <zMax>, respectively.

%

% Requirement: Brain Connectivity Toolbox

% https://sites.google.com/site/bctnet/

% Non-directional matrix 1.

% Mean of z value matrix and its transpose

zMean = (zValue+zValue.')/2;

distZMean = (each component of zMean is inversed);

% Non-directional matrix 2.

% Max of z value matrix and its transpose

zMax = max(zValue,zValue.');

distZMax = (each component of zMax is inversed);

% Betweenness centrality

% Brain Connectivity Toolbox (https://sites.google.com/site/bctnet/) is required

bcDistZMean = betweenness_wei(distZMean)/((N-1)*(N-2));

bcDistZMax = betweenness_wei(distZMax)/((N-1)*(N-2));

%%%%%%%%%%%%%%%%%%%%%%%%%%%%%%%%%%%%%%%%%%%%%

(A-3)

%%%%%%%%%%%%%%%%%%%%%%%%%%%%%%%%%%%%%%%%%%%%%

% Matlab-style code for

% estimation of hierarchical levels of network nodes (S4B Fig)

function [levelOptimized, probabilityOfLevel] = HierarchyPPINetwork(wBin)

% <HierarchyPPINetwork> requires a binarized matrix <wBin>.

% <levelOptimized> is a list of optimized assignments of hierarchical

% levels to each node.

% <probabilityOfLevel> contains the probability with which each node is

% at each level

N = length(wBin);

% All possible assignments of hierarchical levels to nodes

numcand = 0;

for (all possible values of <n1>,<n2>,...,<nN> where <ni> can be any of 1:N)

levelCandidate=[n1,n2,...,nN];

if (all of 1,2,...,max(levelCandidate) are included in <levelCandidate>)

numcand = numcand + 1;

levelCandidate(numcand, :) = levelCandidate;

end

end

% Optimized assignments of hierarchical levels to nodes

% (see Materials and Methods)

numOptimum = 0;

for cand = 1:numcand

cnt = (number of combinations of nodes i and j such that

(wBin(i,j) == 1) &&

(level of node i is lower than that of node j in <levelCandidate(cand,:)>))

if cnt <= 1

numOptimum = numOptimum + 1;

levelOptimized(numOptimum,:) = levelCandidate(cand,:);

end

end

% Probability with which a node is at each level

for node = 1:N

cntLevel = [];

for level = 1:N

cntLevel(level) = (number of <level> in levelOptimized(:,node));

end

probabilityOfLevel(:,node) = cntLevel/numOptimum*100;

end

%%%%%%%%%%%%%%%%%%%%%%%%%%%%%%%%%%%%%%%%%%%%%

(B-1)

%%%%%%%%%%%%%%%%%%%%%%%%%%%%%%%%%%%%%%%%%%%%%

% Matlab-style code for performance prediction analysis (Fig 5B)

function accuracy = performancePrediction(features)

% <performancePrediction> requires <features> involving PPI values

% and task performance labels (high/low), and returns <accuracy> for

% classification accuracy.

% Requirement: LIBSVM

% http://www.csie.ntu.edu.tw/~cjlin/libsvm/

% Demeaning feature values across features.

features = demean(features);

% <crossValidationTest> performs leave-one-out cross-validation on

% experimental sessions, and returns classification accuracy.

% <crossValidationTest> uses LIBSVM for train and test.

% http://www.csie.ntu.edu.tw/~cjlin/libsvm/

accuracy = crossValidationTest(features);

%%%%%%%%%%%%%%%%%%%%%%%%%%%%%%%%%%%%%%%%%%%%%

(B-2)

%%%%%%%%%%%%%%%%%%%%%%%%%%%%%%%%%%%%%%%%%%%%%

% Matlab-style code for predicted impact on performance (Fig 5C)

function predictedImpactOnPerformance = nodeDeletionImpact(features, nodeID)

% <nodeDeletionImpact> requires <features> involving PPI values,

% task performance labels (high/low), and <nodeID>

% indicating the target node to be deleted.

% The function returns <predictedImpactOnPerformance>,

% post-deletion classification accuracy relative to

% pre-deletion classification accuracy.

% Requirement: LIBSVM

% http://www.csie.ntu.edu.tw/~cjlin/libsvm/

% Demeaning.

features = demean(features);

% node-deleted feature set.

nodeDeletedFeatures = nodeDeletion(features, nodeID);

predictedImpactOnPerformance = ...

crossValidationTest(features) - crossValidationTest(nodeDeletedFeatures);

%%%%%%%%%%%%%%%%%%%%%%%%%%%%%%%%%%%%%%%%%%%%%

(B-3)

%%%%%%%%%%%%%%%%%%%%%%%%%%%%%%%%%%%%%%%%%%%%%

% Matlab-style code for performance prediction analysis

% for a larger set of nodes (S14B Fig)

function extendedNodeAnalysis(allFeatures)

% <extendedNodeAnalysis> requires <allFeatures> involving PPI values

% and task performance labels (high/low), and returns <accuracy> for

% classification accuracy.

% Requirement: LIBSVM

% http://www.csie.ntu.edu.tw/~cjlin/libsvm/

% Applying a t-value filter for feature selection

% <tValueFilter> selects <numSelect> features from <allFeatures>

% based on each feature's t-value for the difference in PPIs between

% high vs. low performance sets. Top <numSelect> features are selected.

% Here we select more features <baseFeatures> specified

% by <scaleFactor> for randomization below.

baseFeatures = tValueFilter(allFeatures, numSelect*scaleFactor);

% For each feature selection number <numSelect>, cross validation tests

% are performed for <numRand> times by selecting <numSelect> features

% from <baseFeatures> randomly.

% This procedure aims to reduce fractionation of classification performance

% derived from relative differences in t-values across features.

for i=1:numRand

selectedFeatures=randomSelect(baseFeatures, numSelect);

% Demeaning selected features.

selectedFeatures = demean(selectedFeatures);

% <crossValidationTest> performs leave-one-out cross-validation on

% experimental sessions, and returns classification accuracy.

% <crossValidationTest> uses LIBSVM for train and test.

% http://www.csie.ntu.edu.tw/~cjlin/libsvm/

allAccuracy(i)=crossValidationTest(selectedFeature);

end

% Averaging accuracy across the randomization.

accuracy = mean(allAccuracy);

(B-4)

%%%%%%%%%%%%%%%%%%%%%%%%%%%%%%%%%%%%%%%%%%%%%

% Matlab-style code for predicted impact on performance

% for a larger set of nodes (S14D, E Fig)

function predicatedImpactOnPerformance ...

= nodeDeletionImpactForExtendedNodes(allFeatures, nodeID)

% <nodeDeletionImpactForExtendedNodes> requires <allFeatures>

% involving PPI values, task performance labels (high/low), and <nodeID>

% indicating the target node to be deleted.

% The function returns <predictedImpactOnPerformance>,

% post-deletion classification accuracy relative to

% pre-deletion classification accuracy.

% Requirement: LIBSVM

% http://www.csie.ntu.edu.tw/~cjlin/libsvm/

% Applying a t-value filter for feature selection.

% <tValueFilter> selects <numSelect> features from <allFeatures>

% based on each feature's t-value for the difference in PPIs between

% high vs. low performance sets. Top <numSelect> features are selected.

% Here we select more features <baseFeatures> specified

% by <scaleFactor> for randomization below.

baseFeatures = tValueFilter(allFeatures, numSelect*scaleFactor);

% For each feature selection number <numSelect>, cross validation tests

% are performed for <numRand> times by selecting <numSelect> features

% from <baseFeatures> randomly.

% This procedure aims to reduce fractionation of classification performance

% derived from relative differences in t-values across features.

for i=1:numRand

selectedFeatures=randomSelect(baseFeatures, numSelect);

% Demeaning feature sets.

selectedFeatures = demean(selectedFeatures);

% Deleting target node features.

nodeDeletedFeatures = nodeDeletion(selectedFeatures, nodeID);

% Performing cross validation test.

allPredictedImpactOnPerformance(i) = ...

crossValidationTest(selectedFeatures) ...

- crossValidationTest(nodeDeletedFeatures);

end

% Averaging the predicted impact on performance across the randomization.

predictedImpactOnPerformance = mean(allPredictedImpactOnPerformance);

%%%%%%%%%%%%%%%%%%%%%%%%%%%%%%%%%%%%%%%%%%%%%
